# Radiomics for Detection and Differentiation of Intrahepatic Cholangiocarcinoma: A Systematic Review and Meta-Analysis

**DOI:** 10.3390/cancers18060937

**Published:** 2026-03-13

**Authors:** Zayan Alidina, Illiyun Banani, Umm E. Abiha, Ujala Sultan, Timothy M. Pawlik

**Affiliations:** 1 Medical College, Aga Khan University, Karachi 74800, Pakistan; zayan.alidina@scholar.aku.edu (Z.A.); illiyun.banani@scholar.aku.edu (I.B.); umm.abiha@scholar.aku.edu (U.E.A.); ujala.sultan@scholar.aku.edu (U.S.); 2 Department of Surgery, The Ohio State University Wexner Medical Center and James Comprehensive Cancer Center, Columbus, OH 43210, USA

**Keywords:** intrahepatic cholangiocarcinoma (ICC), radiomics, artificial intelligence (AI), machine learning (ML)

## Abstract

Background: Intrahepatic cholangiocarcinoma (ICC) is an aggressive primary liver malignancy with limited survival, largely due to delayed diagnosis, recurrence and limited effective therapeutic options. Radiomics- and artificial intelligence (AI)-based imaging models have emerged as promising tools to improve noninvasive detection and differentiation of ICC. We conducted a systematic review and meta-analysis to evaluate the diagnostic performance of radiomics-based AI models for ICC. Methods: A systematic search of PubMed, Embase, Scopus, and the Cochrane Library was performed from inception through 2025 in accordance with PRISMA guidelines. Studies assessing radiomics- or AI-based models derived from CT, MRI, PET, or ultrasound for differentiation of ICC from other hepatic lesions were included. Pooled sensitivity, specificity, positive likelihood ratio (PLR), and negative likelihood ratio (NLR) were estimated using a bivariate random-effects model. Study quality and risk of bias were assessed using the Radiomics Quality Score (RQS) and QUADAS-2 tools. Results: Twenty retrospective studies comprising 8746 participants were included. Across pooled validation and test datasets, radiomics-based AI models demonstrated a pooled sensitivity of 0.77 (95% CI, 0.69–0.84) and specificity of 0.88 (95% CI, 0.78–0.94) for differentiating ICC from non-ICC hepatic lesions. The pooled PLR was 6.81 (95% CI, 3.51–13.2), and the pooled NLR was 0.23 (95% CI, 0.09–0.61). CT-based models showed higher diagnostic performance compared with MRI and ultrasound. Subgroup and meta-regression analyses identified imaging modality, contrast phase, segmentation strategy, and validation approach as contributors to interstudy heterogeneity. The overall methodological quality demonstrated a mean Radiomics Quality Score (RQS) of 14.0 (range 11–24), corresponding to approximately 39% of the maximum achievable score. External validation cohorts were incorporated in 60% of the studies, although adherence to standardized feature reproducibility frameworks varied. Conclusions: Radiomics-based AI models demonstrate clinically meaningful diagnostic accuracy for noninvasive differentiation of ICC and may complement conventional imaging in preoperative evaluation. Prospective, multicenter studies with standardized imaging protocols and rigorous external validation are required before routine clinical adoption.

## Simple Summary

Intrahepatic cholangiocarcinoma is a rare but highly aggressive cancer that arises from the bile ducts within the liver and is frequently diagnosed at an advanced stage, limiting treatment options and survival. This study aimed to evaluate whether advanced computer-based analysis of medical images called radiomics, a method that extracts hidden patterns from scans, can improve the detection and differentiation of this cancer from other liver tumors. We analyzed results from 20 studies, including 8746 patients in which artificial intelligence models examined CT, MRI, or ultrasound images. Overall, these models correctly identified the cancer in about 77% of cases and correctly ruled it out in about 88% of cases, with CT-based models showing the strongest performance. These findings suggest that computer-assisted image analysis may support earlier and more accurate diagnosis, helping clinicians make better treatment decisions and potentially improving outcomes for patients with liver tumors.

## 1. Introduction

Intrahepatic cholangiocarcinoma (ICC), the second most common primary liver tumor, is a unique type of cholangiocarcinoma located in the periphery of the liver proximal to the segmental bile ducts [1]. Although ICC is a rare malignancy (comprising only 3% of total gastrointestinal cancers), its incidence has risen from 0.44 to 1.18 cases per 100,000 people between 1973 and 2020 in the United States, corresponding to an annual increase of 2.3% [2]. ICC carries a poor prognosis, with a mortality-to-incidence ratio of 0.95, reflecting its high fatality rate [3,4].

Nearly half of patients with ICC present with advanced disease, rendering them ineligible for curative-intent surgical resection. Five-year survival is 25% for localized disease but drops to 2% for distant disease, underscoring the importance of early detection [2]. Despite advances in imaging, early diagnosis remains challenging, as CT and MRI have limitations in differentiating subtle imaging features and distinct lesion phenotypes on routine scans. While tumors with typical features are easily detected, masses with atypical features such as central necrosis are difficult to differentiate pre-operatively from hepatocellular carcinoma, immature abscesses, metastases, hepatic tuberculosis and other tumors with abundant fibrous stroma, which may demonstrate overlapping enhancement patterns and morphological patterns [5]. This diagnostic ambiguity often results in delayed recognition of ICC, postponing appropriate surgical or systemic management and adversely affecting prognosis. Ultrasound, including contrast-enhanced ultrasound (CEUS), is limited by operator dependence [6,7]. Accurate staging is also difficult because CT and MRI have low sensitivity for lymph node invasion, while qualitative interpretation contributes to diagnostic variability and delay [6].

By applying quantitative analysis on medical images, radiomics generates high-dimensional data, which has transformed the landscape of disease diagnosis and prognosis. Intricate imaging features that may be overlooked during conventional imaging can be more reliably captured using objective, algorithm-based approaches, reducing subjectivity and improving diagnostic consistency [8]. Image acquisition, region of interest segmentation, quantitative feature extraction (such as texture, shape, intensity, and wavelet-based metrics), and machine learning model analysis to determine patterns linked to disease characteristics are the usual steps in the process [9]. This makes it possible to predict tumor behavior, identify subtle phenotypic differences, and stratify risks in a manner not achievable with conventional qualitative imaging interpretation.

Individual studies have reported a higher diagnostic accuracy of AI-based models to differentiate ICC from hepatocellular carcinoma and other liver lesions compared with conventional imaging assessment. CT- and MRI-based radiomics models have reported AUCs exceeding 0.85–0.90 for ICC and hepatocellular carcinoma (HCC) discrimination in multiple cohorts [10,11]. Deep learning approaches, particularly convolutional neural networks (CNNs), further improve performance by automatically learning hierarchical spatial and textural features beyond handcrafted radiomics, thereby minimizing diagnostic delays and improving prognosis.

Despite the potential of radiomics and AI to enhance ICC detection, differentiation and prognosis, most current research is based on small, single-center, and methodologically diverse data with differences in imaging protocols, feature extraction, and model validation. Most earlier reviews have not focused on ICC-specific imaging characteristics, instead grouping ICC with other primary liver or biliary tract malignancies, thereby obscuring distinct radiologic patterns and disease-specific diagnostic challenges. Therefore, the objective of the current study was to address this gap by quantitatively summarizing diagnostic accuracy, prognostic value, and study quality across available radiomics and AI models. This systematic review and meta-analysis aimed to evaluate radiomics-based models derived from CT, MRI, PET, or ultrasound for the early detection and differentiation of ICC patients. Subgroup analyses by imaging modality, patient cohort, and model composition (radiomics-only versus combined clinical models) delineated sources of heterogeneity and highlighted the potential for clinical translation.

## 2. Methods

A systematic review was conducted that adhered to the Preferred Reporting Items for Systematic Reviews and MetaAnalyses (PRISMA) report [12]. The protocol was registered at the PROSPERO platform under the ID CRD420261306635. The review aligned with the Cochrane Handbook for Systematic Reviews of Diagnostic Test Accuracy. The primary objective was to evaluate the diagnostic performance of radiomics-based models derived from cross-sectional imaging modalities for the detection and differentiation of intrahepatic cholangiocarcinoma (ICC) from non-ICC liver cancer.

### 2.1. Inclusion and Exclusion Criteria

Eligibility criteria were structured according to diagnostic test accuracy principles consistent with the Cochrane Handbook, incorporating elements of the PITROS framework: Population (P): adult participants (≥18 years) with a histopathologically confirmed diagnosis of ICC who underwent imaging with cross-sectional imaging modalities. Index Test (I): machine learning (ML) techniques (such as deep learning, support vector machine (SVM) or hybrid techniques) used in radiomic models for ICC differentiation using CT, MRI, PET, or ultrasound images. Target Condition (T): ICC. Reference Standard: histopathology. Outcomes (O): diagnostic performance metrics including sensitivity, and specificity. Study design (S): original research articles (observational studies, cohort studies including retrospective or prospective analysis or case–control studies, or clinical trials) with no restrictions on the year of publication.

Exclusion criteria included (1) studies without a clear eligibility criteria, including reports limited to non-neoplastic liver diseases (e.g., hepatitis, Budd–Chiari syndrome, hemochromatosis or Wilson disease); (2) studies including only model training without independent validation or testing cohorts; (3) studies addressing only prognosis, treatment response, or survival prediction; (4) multiple classification articles or publications not reporting diagnostic performance measures; and (5) conference abstracts, editorials, letters, or reviews.

### 2.2. Information Sources and Search Strategy

A comprehensive search strategy was developed in accordance with diagnostic test accuracy principles. The search incorporated three core components: (1) radiomics and artificial intelligence terminology, (2) intrahepatic-cholangiocarcinoma-specific terms, and (3) diagnostic performance terminology (e.g., differentiation, classification, discrimination, accuracy). Prognostic-only terminology (e.g., survival, recurrence, treatment response) was excluded to minimize conceptual heterogeneity and ensure alignment with the study objective of diagnostic differentiation.

Databases searched included PubMed, Embase, Scopus, and Cochrane CENTRAL from inception through December 2025. The complete revised search strategies are presented in the Supplementary Materials.

### 2.3. Study Selection

**Figure 1:**
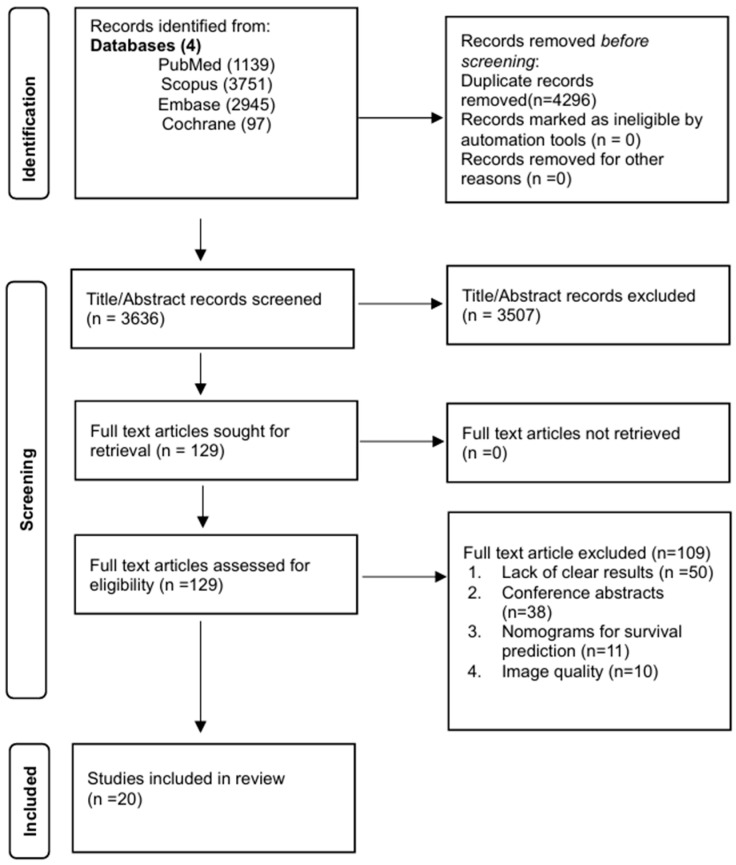
Preferred Reporting Items for Systematic Reviews and Meta-Analyses (PRISMA) flow diagram.

All articles retrieved from the systematic search were exported to the Rayyan software (Version 1.4.3) for initial deduplication. Two authors (U.A., U.S.) independently reviewed the titles and abstracts of articles to assess relevance against the eligibility criteria. Full texts of the shortlisted articles were read to ensure eligibility to inclusion and exclusion criteria. A third author (ZA) resolved any discrepancies at each stage. The full texts of the potentially relevant studies were retrieved and evaluated independently by two reviewers, and discrepancies were resolved by consulting a third reviewer. Figure 1 represents the PRISMA flowchart for the selection and screening process.

The two primary reviewers (U.A. and U.S.) were medical students with formal research training and over three years of structured experience in systematic reviews and evidence synthesis. The third reviewer (Z.A.), who adjudicated discrepancies, was a final-year medical student with more than two years of research experience in hepatobiliary malignancies and prior authorship of radiomics-based meta-analyses. Discrepancies during title/abstract and full-text screening occurred in approximately 12% of cases and were resolved through consensus discussion; a third reviewer was required for adjudication in 6% of screened studies.

### 2.4. Data Extraction

Data were extracted from each article by two independent reviewers and compiled by a third reviewer. The same two reviewers (U.A. and U.S.) performed data extraction independently. Both had prior experience in radiomics literature appraisal and quantitative evidence synthesis. Disagreements occurred in approximately 9% of extracted variables and were resolved through joint review, with third-reviewer adjudication required in fewer than 5% of instances. For each study, information on study characteristics was included, including the first author’s name, publication year, country, study design, and recruitment period. Population and imaging details were also extracted such as sample size, sex distribution, comparator group, imaging modality, region of interest (ROI), segmentation approach, feature extraction tools, and image preprocessing or specifications. Furthermore, the model names and corresponding machine learning techniques used to develop the radiomic models were recorded. In addition, performance measures such as sensitivity, specificity, and overall diagnostic accuracy were extracted. Validation strategies, both internal and external, as well as any use of a prospective cohort, were also noted to assess pre-diagnostic clinical utility. If a study included models with separate training, validation, and testing datasets, only the results from the testing dataset were extracted for meta-analysis. Any inconsistencies in extracted data were resolved through reviewer discussions.

### 2.5. Quality Assessment

Two independent reviewers (Z.A and I.B) conducted both evaluations for quality assessment. The Radiomics Quality Score (RQS) was used to evaluate the methodological rigor of the final list of studies [13]. Both had formal training in diagnostic test accuracy methodology and prior experience applying the QUADAS-2 and RQS frameworks. Discrepancies in quality scoring were observed in approximately 15% of domain-level judgments and were resolved by structured consensus; third-reviewer (T.P) adjudication was required in 7% of cases. Meanwhile, the Quality Assessment of Diagnostic Accuracy Studies (QUADAS-2) was utilized to assess the risk of bias and study applicability among the included studies [14]. Discrepancies in RQS and QUADAS-2 scoring were resolved via mutual consensus and reviewer discussion. The results for both evaluations are provided in the Supplementary Materials.

### 2.6. Meta-Analysis

All quantitative analyses were conducted using the OpenMeta analyst and Cochrane RevMan 5.4.1 software (Review Manager Version 5.4.1, the Cochrane Collaboration, Copenhagen, Denmark) for formation of forest plot and HSROC curves. For each study, the extracted data comprised the sample sizes of the experimental (cases) and comparator (controls) groups, along with reported sensitivity and specificity, which were utilized to construct 2 × 2 contingency tables comprising true positives (TP), false positives (FP), false negatives (FN), and true negatives (TN). For studies that did not provide these diagnostic metrics, these values were calculated using specificity and sensitivity results provided within the study. Subsequently, estimates of diagnostic performance metrics, sensitivity, and specificity, with corresponding 95% confidence intervals were reviewed and calculated.

A meta-analysis was conducted using bivariate random-effects model to adjust for any possible correlations between sensitivity and specificity. Importantly, pooling was conducted at the level of reported diagnostic performance (sensitivity, specificity) and not at the feature level. Given intrinsic differences in radiomic feature derivation across CT, MRI, ultrasound, and PET, modality-specific subgroup analyses and meta-regression were performed to evaluate heterogeneity attributable to imaging modality. The DerSimonian–Laird method was employed to estimate the pooled sensitivity and specificity. Heterogeneity was assessed between studies using the I^2^ statistic and Cochran’s Q test, with values above 50% indicating substantial heterogeneity. Moreover, to minimize the potential bias among the studies, diagnostic log odds ratios (DORs) were calculated for each study and incorporated into the meta-regression analyses to examine potential sources of heterogeneity. Sensitivity analysis was conducted using leaving-one-out study analysis.

## 3. Results

### 3.1. Study Selection and Characteristics

The initial systematic literature search yielded a total of 7932 records. Following the removal of 4296 duplicate publications, 3636 unique records underwent title and abstract screening. Subsequently, 129 full-text articles were rigorously assessed for eligibility against the predefined inclusion and exclusion criteria. This process culminated in the inclusion of 20 studies for qualitative synthesis in the meta-analysis. The PRISMA flow diagram detailing the selection process is provided in Figure 1. The most frequent reasons for exclusion at the full-text stage were a lack of clear diagnostic performance metrics (*n* = 50), publication as conference abstracts (*n* = 38), a focus on nomograms for prediction and detection of ICC (*n* = 11), and studies aimed primarily at improving image quality (*n* = 10).

The key characteristics of the included studies are summarized in the accompanying Table 1. All 20 studies employed a retrospective design and were published between 2018 and 2025, with data originating from institutions in China (*n* = 16) [7,15,16,17,18,19,20,21,22,23,24,25,26,27,28,29], the United States of America (*n* = 2) [30,31], Germany (*n* = 1) [32], and Japan (*n* = 1) [33]. The comparator groups across all studies primarily comprised patients with hepatocellular carcinoma (*n* = 12) [15,16,17,21,22,23,24,25,26,27,31,32], while few studies had a comparator group that comprised various liver tumors (involving other benign and malignant liver tumors and metastasis) (*n* = 4) [7,18,19,20]; other studies had a comparator group consisting of patients with inflammatory liver mass with hepatolithiasis [27,28,29]. Data sources were heterogeneous, ranging from single-center institutional studies to multi-institutional collaborations. The scale of data used for model development varied substantially with the size of training sets, ranging from 112 to 493 patients. The reference standard for definitive ICC diagnosis in all included studies was histopathological confirmation (either from resection or biopsy). Regarding imaging technique, 10 studies utilized contrast-enhanced computed tomography (CT), while 2 studies utilized non-contrast CT scan. Furthermore, four studies reported using magnetic resonance imaging (MRI) and two studies utilized ultrasound for differentiation and detection of ICC. A single study conducted by Cheng et al. validated a model using both radiomics models that comprised non-contrast CT and MRI scans. Most studies primarily used both the portal and arterial venous phase (*n* = 7), non-contrast phase (*n* = 6), arterial phase only (*n* = 5), and portal venous phase (*n* = 2).

The number of patients with ICC was 1588 (18.2%), while the other 3119 (35.7%) patients had other liver tumor/masses including unspecified liver lesions (46.1%). A single study conducted by Midya et al. reported the use of 814 scans, among which 246 scans comprised patients with ICC, while 568 scans involved patients with various liver tumors, including HCC, colorectal liver metastasis (CRLM), and benign liver tumors [31]. There was a greater proportion of males across all studies (male: *n* = 2206, 73.3%; female: *n* = 803, 26.7%), with a mean participant age ranging from 53.9 ± 12.4 years to 67.9 ± 10.1 years among patients with an ICC. Data sources were primarily extracted from single-center institutional records (*n* = 14), while other studies were extracted from multicenter institutional datasets (*n* = 6) (Supplementary Table S1). Twelve studies (60.0%) had external validation datasets.

### 3.2. Study Participants and Algorithm Characteristics

A diverse spectrum of artificial intelligence and machine learning models were employed to diagnose and differentiate ICC. Convolutional neural network (CNN) classifiers were the most frequently utilized architecture, which were featured in nine studies, followed by radiomic models involving logistic regression in eight studies, and support vector machines (SVMs) in two studies. Wang et al. integrated the use of both CNN and SVM models to differentiate ICC [21]. A sophisticated, custom-built cascaded deep learning network known as AutoML Programming was utilized in a study conducted by Hu et al. [23]. The methodology was predominantly rooted in radiomics, involving the high-throughput extraction of quantitative features from medical images. Image segmentation was performed manually by radiologists or researchers in 16 studies, whereas segmentation was done automatically using deep learning models in 4 studies. Common preprocessing steps included intensity normalization, windowing, and data augmentation techniques to improve model robustness. The validation strategies adopted were multifaceted, encompassing internal validation via random data splits or k-fold cross-validation and external validation on completely independent datasets, with the latter being employed in eight studies. A summary of the algorithms and model characteristics is reported in Table 2.

### 3.3. Quality Analysis

**Figure 2:**
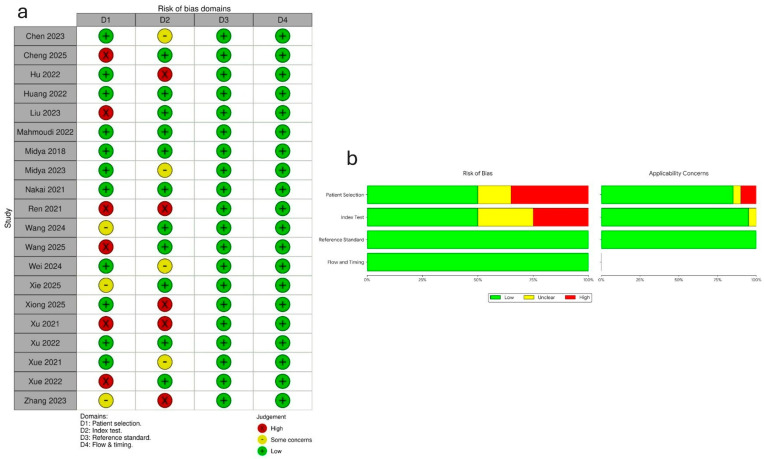
(**a**) Table representing QUADAS-2 scores of individual studies for each domain [7,15,16,17,18,19,20,21,22,23,24,25,26,27,28,29,30,31,32,33]. (**b**) Graph representing proportions of judgements across each domain.

The methodological quality of the 20 included studies was characterized by a high degree of consistency in reference standards and procedural flow, although marked variability was observed in patient selection and index test protocols. According to the QUADAS-2 assessment, all studies (100%) demonstrated a low risk of bias in the Reference Standard (D3) and Flow and Timing (D4) domains. However, 30% (*n* = 6) of studies were flagged as having high risk for Patient Selection (D1) and 25% (*n* = 5) for Index Test (D2) bias. Notably, the studies by Ren et al. and Xu et al. were identified as having high risk across both D1 and D2, suggesting potential vulnerabilities in their recruitment strategies and test interpretations [20,29]. The QUADAS 2 scores are represented in Figure 2.

Complementing the risk of bias assessment, the Radiomics Quality Score (RQS) revealed a broad spectrum of radiomics-specific rigor. The scores ranged from a minimum of 11 to a maximum of 24, with a mean score of approximately 14.0. The study by Wei et al. achieved the highest score (RQS = 24), indicating superior adherence to standardized radiomics reporting and validation workflows [20]. In contrast, several studies scored at the lower end of the spectrum with an RQS of 11 [15,17,21]. Interestingly, while some high-risk QUADAS-2 studies maintained moderate RQS scores (e.g., Ren 2021 with an RQS of 15) [26], the overall trend suggested that more recent studies, such as Wei [20], prioritized both clinical diagnostic validity and radiomics technical reproducibility.

### 3.4. Meta-Analysis

**Figure 3:**
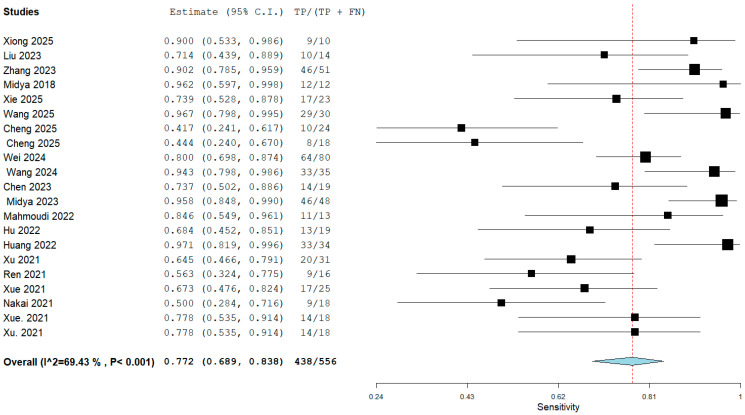
Forest plot with pooled sensitivity [7,15,16,17,18,19,20,21,22,23,24,25,26,27,28,29,30,31,32,33]. In the forest plot, black squares represent the sensitivity estimate of each study, with square size proportional to study weight, and **horizontal lines** indicate the 95% confidence intervals. The red dotted vertical line represents the pooled sensitivity estimate. The blue diamond indicates the overall pooled sensitivity, with its width representing the 95% confidence interval.

**Figure 4:**
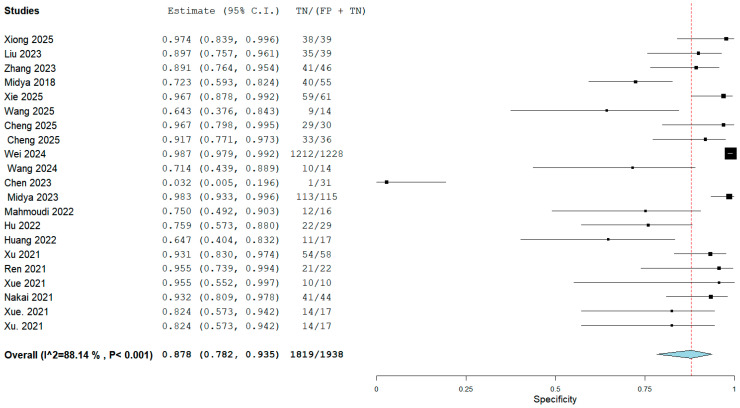
Forest plot with pooled specificity [7,15,16,17,18,19,20,21,22,23,24,25,26,27,28,29,30,31,32,33]. In the forest plot, black squares represent the sensitivity estimate of each study, with square size proportional to study weight, and **horizontal lines** indicate the 95% confidence intervals. The red dotted vertical line represents the pooled sensitivity estimate. The blue diamond indicates the overall pooled sensitivity, with its width representing the 95% confidence interval.

**Figure 5:**
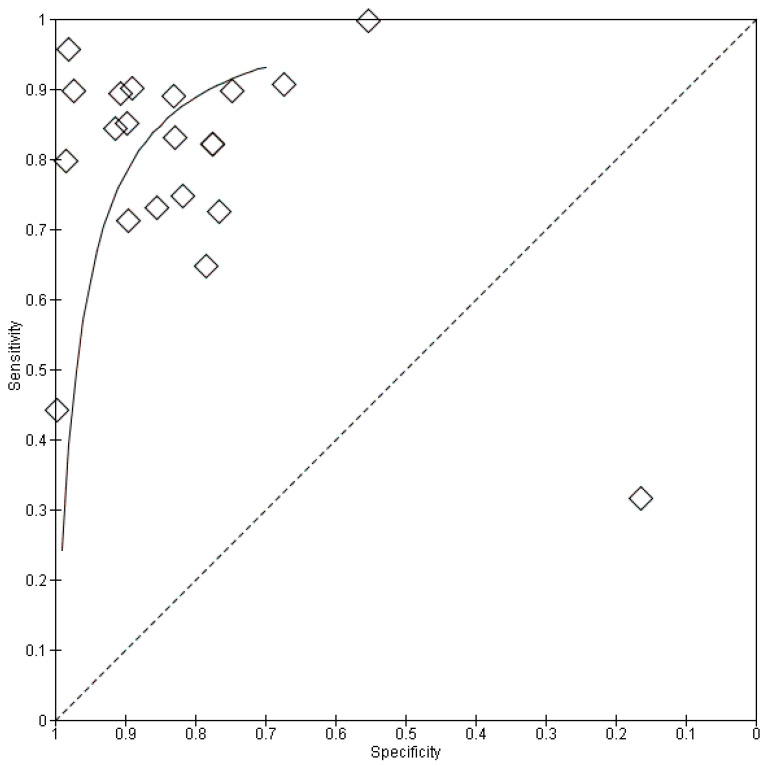
SROC plot of diagnostic performance of artificial intelligence machine learning models for differentiation in ICC patients. Diamond markers represent the paired sensitivity and specificity values reported by individual studies included in the meta-analysis, illustrating the distribution of diagnostic performance across studies in the SROC space.

The meta-analysis incorporated 20 studies encompassing a total of 8746 participants and 814 scans. For the meta-analysis, data from the selected test or validation datasets resulted in 2330 individual participants with 161 scans, which were pooled. Cheng et al. utilized both CT and MRI to differentiate ICC and reported individual results for each modality [19]. The results for both modalities were used in a pooled analysis of diagnostic metrics that comprised sensitivity, specificity, and subgroup analysis. The sample size of the individual studies demonstrated considerable variation, ranging from 35 to 1308 participants. A bivariate random-effects meta-analysis was performed on the 20 included studies, utilizing data from their respective test datasets (local, external, test, or validation sets) to derive pooled estimates of diagnostic performance. The analysis demonstrated that AI models achieved a pooled sensitivity of 0.77 (95% confidence interval [CI]: 0.69–0.84; I^2^:69.4%) and a pooled specificity of 0.88 (95% CI: 0.78–0.94; I^2^: 88.1%) to differentiate ICC. The pooled sensitivity and specificity results are noted in Figure 3 and Figure 4. The pooled positive likelihood ratio (PLR) was 6.81 (95% CI, 3.51–13.2; I^2^: 93.1%); however, the negative likelihood ratio (NLR) was 0.23 (95% CI, 0.09–0.61; I^2^: 96.9%). The forest plot for pooled PLR and NLR is reported in Supplementary Figure S1. The interstudy heterogeneity level was high in all groups (all *p* values < 0.01). Although substantial heterogeneity was observed, no single study disproportionately influenced pooled estimates on leave-one-out sensitivity analysis, suggesting relative robustness of the aggregated results despite methodological diversity. Furthermore, the hierarchical summary receiver operating characteristic (HSROC) curves for the AI algorithms are illustrated in Figure 5.

Meta-regression was used to investigate the effect of variables, including type of AI algorithm, validation, type of diagnostic modality, contrast phases, selected dataset, and segmentation, in the analysis of diagnostic modalities. Studies involving manual data segmentation reported greater sensitivity versus studies with automated data segmentation. Furthermore, decreased sensitivity was also observed in studies with combined arterial and venous phase contrast and non-contrast versus studies with portal venous phase; however, there were no significant results compared with arterial phase contrast studies. For other variables such as AI algorithm, validation, selected dataset and diagnostic modality, no significant results were obtained during meta-regression related to sensitivity. In contrast, ultrasound had decreased specificity compared with CT scan to differentiate ICC. In terms of AI algorithm, contrast phase, selected dataset and segmentation, no results were associated with improved specificity on the meta-regression. To evaluate the possible confounding role of interstudy heterogeneity, a sensitivity analysis was performed based on the leave-one-out method (Supplementary Figure S2).

### 3.5. Subgroup Analysis

Pre-specified subgroup analyses were conducted based on the type of dataset used to evaluate the clinical timing of the patient cohort. In the analysis stratified by dataset type, models evaluated on local test sets (nine studies) had a pooled sensitivity of 0.77 (95% CI: 0.59–0.88) and a pooled specificity of 0.87 (95% CI: 0.68–0.95). In contrast, models evaluated on validation test sets (six studies) reported a pooled sensitivity of 0.82 (95% CI: 0.66–0.931) and pooled specificity of 0.87 (95% CI: 0.74–0.95). A third subgroup, comprising studies that reported performance on a dedicated external test set (five studies), yielded pooled sensitivity of 0.72 (95% CI: 0.59–0.82) and specificity of 0.78 (95% CI: 0.69–0.85). Increased heterogeneity was reported in subgroup analysis stratified by type of dataset, except for external test set, which reported reduced heterogeneity (I^2^: 21.2%). The forest plots for sensitivity and specificity are reported in Supplementary Figure S3. Furthermore, plots for PLR and NLR are provided in Supplementary Figure S4.

When stratified by type of validation, for the internal validation cohort (12 studies), the AI models achieved a pooled sensitivity of 0.81 (95% CI: 0.67–0.89) and a pooled specificity of 0.84 (95% CI: 0.71–0.92). For the external validation cohort (eight studies), the models demonstrated a pooled sensitivity of 0.76 (95% CI: 0.65–0.85) and specificity of 0.92 (95% CI: 0.77–0.97). Increased heterogeneity was reported in subgroup analysis stratified by type of validation. The forest plot for sensitivity and specificity is reported in Supplementary Figure S5. Plots for PLR and NLR are provided in Supplementary Figure S6.

In a subgroup analysis related to AI classifier, most studies used AI models that involved CNN and radiomic classifiers, including logistic regression and machine learning. For the CNN classifier, the pooled sensitivity was 0.77 (95%CI: 0.60–0.88), while the reported pooled specificity was 0.91 (95%CI: 0.73–0.97). In contrast, for the radiomic model, the reported pooled sensitivity was 0.80 (95%CI: 0.70–0.87), while the pooled specificity was 0.83 (95%CI: 0.71–0.90); however, the results were not significant (*p* = 0.111). Increased heterogeneity was reported in subgroup analysis stratified by AI classifier. The forest plot for sensitivity and specificity is reported in Supplementary Figure S7. Furthermore, plots for PLR and NLR are provided in Supplementary Figure S8.

In the analysis stratified by diagnostic modality, models utilizing CT scans (13 studies) demonstrated a pooled sensitivity of 0.79 (95% CI: 0.68–0.87) and a pooled specificity of 0.91 (95% CI: 0.81–0.96). In contrast, models integrating an MRI modality (six studies) reported a pooled sensitivity of 0.78 (95% CI: 0.59–0.90) and a pooled specificity of 0.85 (95% CI: 0.72–0.92). A third subgroup, comprising studies that reported performance using US scans (two2 studies), yielded a pooled sensitivity of 0.65 (95% CI: 0.47–0.80) and specificity of 0.46 (95% CI: 0.002–0.99). Increased heterogeneity was reported in subgroup analysis stratified by diagnostic modality, except for the US modality, which reported reduced heterogeneity (I^2^: 13.3%). The forest plots for sensitivity and specificity are reported in Supplementary Figure S9, and the plots for PLR and NLR are provided in Supplementary Figure S10.

When stratified by type of segmentation, the AI models achieved a pooled sensitivity of 0.73 (95% CI: 0.63–0.81) and a pooled specificity of 0.87 (95% CI: 0.81–0.92) for the manual segmentation cohort (16 studies). For the automated segmentation cohort (eight studies), the models demonstrated a pooled sensitivity of 0.87 (95% CI: 0.76–0.93) and specificity of 0.85 (95% CI: 0.43–0.98) (*p* = 0.057). Increased heterogeneity was reported in subgroup analysis stratified by type of segmentation. The forest plot for sensitivity and specificity is reported in Supplementary Figure S11, and the plots for PLR and NLR are provided in Supplementary Figure S12.

## 4. Discussion

The current meta-analysis demonstrated that radiomics- and artificial intelligence (AI)-based imaging approaches yielded moderate-to-high diagnostic accuracy to differentiate ICC from other hepatic lesions. Radiomics has been increasingly investigated across a broad spectrum of oncologic applications, including lung, colorectal, pancreatic, breast, and hepatocellular carcinomas, demonstrating value in tumor detection, subtype differentiation, treatment response prediction, and survival stratification [8,34]. Related to liver malignancies specifically, radiomics has been used to predict microvascular invasion, tumor grade, and recurrence risk related to HCC [35,36]. Radiomics has been demonstrated to be a useful noninvasive imaging biomarker that complements conventional radiologic interpretation for HCC [35,36]. The application of radiomics to patients with ICC has remained relatively underexplored and methodologically heterogeneous, with most prior reports grouping ICC with other primary liver tumors rather than addressing its distinct biological and radiologic phenotype [4,37]. The current study was important because we specifically examined the role of radiomics among patients with ICC.

The pooled sensitivity (0.77) and specificity (0.88) indicated a clinically meaningful capacity for radiomics models to enhance noninvasive characterization of liver tumors, addressing a persistent challenge in surgical oncology, in which conventional imaging often lacks precision [11,32,38]. It is important to emphasize that pooling was performed at the level of diagnostic accuracy metrics rather than combining radiomic features across imaging modalities. Because CT, MRI, and ultrasound generate modality-specific quantitative features, direct feature comparability was not assumed. Instead, pooled estimates reflected aggregate clinical performance of modality-specific models, with subgroup analyses demonstrating modality-dependent variability. While traditional imaging criteria rely on subjective radiologist interpretation, radiomics enables quantitative assessment of tumor phenotypes, capturing subvisual heterogeneity linked to cellular density, stromal structure, and vascular architecture [11,18,39]. These imaging biomarkers may therefore serve as surrogates of biological aggressiveness and contribute to improved diagnostic confidence.

Beyond diagnostic discrimination, radiomic signatures likely reflect core pathophysiological mechanisms underlying ICC biology. ICC is characterized by a dense desmoplastic stroma, heterogeneous angiogenesis, and frequent molecular alterations such as IDH1/2 mutations and FGFR2 fusions. These features generate spatial heterogeneity that is visually appreciable on multiphasic CT and MRI and quantifiable through texture-based radiomics. Radiologic–pathologic correlation studies have demonstrated that delayed enhancement and peripheral rim patterns correspond to fibrosis distribution and tumor cellularity [5]. Texture features such as entropy and kurtosis may therefore serve as imaging surrogates for stromal density, microvascular architecture, and intratumoral complexity—factors known to influence recurrence risk and systemic therapy responsiveness. Meta-analytic data further suggest that portal-venous-phase radiomics captures these vascular–stromal interactions with higher sensitivity [11].

Emerging radiogenomic evidence also supports a biological basis for these quantitative imaging markers. Molecularly defined ICC subtypes, particularly IDH-mutant and FGFR2-rearranged tumors, demonstrate distinct microenvironmental and metabolic profiles that may translate into measurable imaging phenotypes [2]. From a therapeutic standpoint, these associations are increasingly relevant as targeted therapies and immunotherapy become integrated into first-line management of advanced biliary tract cancers. Radiomic features reflecting peritumoral heterogeneity and vascular complexity may also correlate with microvascular invasion and lymph node metastasis, directly influencing surgical planning and risk stratification [4]. Thus, radiomics should be interpreted not merely as a classification tool but as a noninvasive imaging biomarker platform anchored in tumor biology and increasingly relevant to precision surgical oncology.

Consistent with earlier reports on radiomics, the results from the current study underscore the translational potential of radiomics in the clinical workflow [4,11,38]. For instance, Wang et al. reported that CT radiomics was better able to differentiate ICC from HCC with relatively high accuracy, while MRI radiomics models have demonstrated robust performance across external validation cohorts [4,11]. Likewise, AI-augmented MRI models using advanced network architectures can reliably differentiate ICC subtypes and improve diagnostic accuracy compared with conventional imaging criteria [40,41]. Recent hybrid studies indicate that multiparametric radiomics combining clinical, biochemical, and imaging features might yield even higher predictive power. This multi-factor approach reflects the complex interplay of tumor biology and host factors that drive cancer behavior and improves model robustness across heterogeneous populations [34,37].

In the subgroup analysis, CT-based radiomics consistently achieved higher performance metrics compared with MRI and ultrasound. This finding aligns with the literature, which has noted that stability of CT features across institutions and the high contrast resolution of portal venous phase imaging can enhance lesion conspicuity [11,37,38]. Meta-regression demonstrated that models based on portal venous phase imaging were associated with improved sensitivity, likely because this phase better captures tumor microvascular patterns critical for ICC characterization. While MRI offers superior soft-tissue contrast, variability in acquisition parameters (field strength, sequences, contrast timing) can undermine feature reproducibility. Nevertheless, advanced MRI-based radiomics models, particularly those approaches employing diffusion-weighted imaging and dynamic contrast enhancement, have demonstrated excellent discrimination when rigorously standardized [41,42]. Ultrasound-based radiomics, while promising for point-of-care assessment, are generally inferior to CT and MRI, likely owing to operator variability and limited spatial resolution [43].

The meta-regression findings indicated no clear superiority for a specific classifier algorithm, whether traditional machine learning (e.g., support vector machines, random forests) or deep neural networks were utilized. However, deep learning approaches, especially convolutional neural networks (CNNs), often demonstrated higher specificity, suggesting potential advantages to reduce false positives in various clinical settings [41,44,45]. Segmentation strategy emerged as an important source of heterogeneity. Models using manual segmentation demonstrated higher sensitivity but also greater variability, reflecting expert delineation of tumor boundaries at the expense of scalability. In contrast, semi-automated and fully automated segmentation algorithms improved reproducibility, although the decreased sensitivity suggests that further refinement of these tools is necessary before widespread clinical deployment [11,18,45]. The meta-regression also elucidated key contributors to performance variability across studies. Beyond modality and phase effects, differences in preprocessing pipelines (normalization, resampling), feature reduction methods, population demographics, and institutional imaging protocols all contributed to heterogeneity. This finding underscores the need for standardized radiomics reporting frameworks such as the Image Biomarker Standardization Initiative (IBSI) and TRIPOD guidelines for diagnostic model transparency [46,47,48]. Notably, studies with external validation datasets tended to have modestly reduced accuracy compared with internal or split-sample validation, highlighting the risk of overfitting and the necessity for multicenter model testing prior to clinical application [32,42].

Radiomics signatures have been associated with histopathological features that influence prognosis, including microvascular invasion, stromal desmoplasia, and tumor grade [35,36,49,50]. For example, models incorporating peritumoral radiomic features have the ability to predict lymph node metastasis and microvascular invasion with promising accuracy, offering noninvasive biomarkers that may inform surgical planning and prognostication. Importantly, integration of radiomics with molecular and genomic profiling (i.e., radiogenomics) may further enhance personalized care. Early work suggests that imaging phenotypes correlate with mutational landscapes (e.g., IDH1/2, FGFR2 fusions), which may have implications for targeted therapy selection and clinical trial stratification [51,52]. From a surgical oncology viewpoint, accurate preoperative tumor classification influences decisions regarding extent of resection, lymphadenectomy, transplantation eligibility, and neoadjuvant therapy planning. Radiomics-based decision support tools have the potential to streamline multidisciplinary workflows, reduce diagnostic uncertainty, and minimize unnecessary biopsies or diagnostic procedures [40,42]. Furthermore, radiomics may aid in longitudinal monitoring of treatment response, recurrence surveillance, and risk stratification for adjuvant therapy decisions; areas in which conventional imaging and serum markers are often insufficient.

Despite theoretical advantages, radiomics-based decision support tools have not yet achieved widespread implementation in routine hepatobiliary practice. Several methodological and translational barriers persist. Substantial variability in imaging acquisition protocols, reconstruction parameters, segmentation strategies, and feature extraction pipelines limit reproducibility and cross-institutional generalizability [42,47]. The need for standardized feature definitions and harmonized preprocessing has been emphasized by the Image Biomarker Standardization Initiative (IBSI), yet adherence remains inconsistent across studies. In addition, many machine learning and deep learning models function as “black box” systems with limited interpretability, raising concerns regarding transparency, clinician trust, and regulatory oversight [20,45]. Retrospective designs, modest sample sizes, and limited prospective external validation increase the risk of overfitting and performance optimism, generating concerns highlighted in the predictive modeling literature [46,48].

Despite these hurdles, limitations regarding the use of AI continue to be progressively addressed. Increasing multicenter collaborations and structured reporting standards such as TRIPOD-AI and CONSORT-AI aim to improve methodological transparency and external validity [46,49]. Harmonization techniques, radiogenomic integration, and explainable AI approaches are also emerging as strategies to enhance robustness and biological interpretability in assessing liver malignancies [51,52]. In the specific context of ICC, in which preoperative diagnostic uncertainty directly impacts surgical resectability assessment, lymphadenectomy planning, and systemic therapy sequencing, the clinical need for reliable noninvasive differentiation remains substantial. As prospective validation studies expand and technical standardization improves, radiomics models are likely to transition from exploratory research tools to structured adjuncts within multidisciplinary tumor board decision-making, particularly in high-volume hepatobiliary centers.

The current study benefited from a robust methodological approach, including a comprehensive literature search, quantitative meta-analysis, and meta-regression to identify performance modifiers. Quality assessment with QUADAS-2 and the Radiomics Quality Score (RQS) provided an evidence-based appraisal of study design and potential bias. By focusing exclusively on ICC and focusing on modality, segmentation, and classifier effects, the findings advance the field beyond broad reviews of mixed hepatic malignancies and positions radiomics as a clinically meaningful imaging biomarker [4,11]. There were, however, several limitations. Retrospective reports and single-center datasets predominate, increasing susceptibility to selection bias. In addition, imaging heterogeneity, including differences in scanner models, contrast protocols, and field strength, may have limited direct comparison and model generalization. Persistent heterogeneity likely reflected variability in imaging acquisition protocols, feature extraction pipelines, comparator lesion types, and retrospective study designs. Limited statistical power within subgroups may have reduced the ability of the meta-regression to detect significant covariate effects. Many of the included studies also lacked rigorous external validation, which can adversely impact performance metrics and result in overfitting when models are not tested across different institutions. While radiomics may correlate with molecular features, prospective validation in diverse cohorts is needed to confirm these relationships and fully realize their translational potential. Nevertheless, the accumulation of evidence, including external validations, multicenter cohorts, and deep learning models, strengthens confidence in the utility of radiomics for ICC and highlights a clear trajectory toward clinical implementation.

## 5. Conclusions

In conclusion, this systematic review and meta-analysis demonstrated that radiomics- and artificial-intelligence-based imaging models were associated with robust and clinically meaningful performance in the noninvasive differentiation of ICC from other hepatic malignancies. By synthesizing evidence across diverse imaging modalities, analytical pipelines, and validation strategies, the study highlights the substantial potential of quantitative imaging biomarkers to complement conventional radiological assessment and enhance preoperative decision-making. The findings underscore the importance of imaging phase selection, segmentation methodology, and rigorous validation to optimize diagnostic accuracy. Moreover, the emerging association between radiomic signatures and tumor biology reinforce the translational relevance of these tools for personalized risk stratification and treatment planning. Despite persistent methodological heterogeneity and limited prospective validation, the growing body of evidence supports the integration of radiomics into multidisciplinary care pathways. Future multicenter, prospective studies incorporating standardized protocols, radiogenomic correlations, and cost-effectiveness analyses are warranted to facilitate regulatory approval and routine clinical implementation.

## Figures and Tables

**Table 1:** Summary of the main characteristics of the studies.

Study	Year	Country	Study Design	Time Period	Comparator	Data Sets	No. of Images per Training Set	No. of Images per Validation Set	No. of Images per Local Test Set	Radiomics/Contrast	Reference Standard
Xiong et al. [15]	2025	China	Retrospective Case–Control	2015–2020	HCC patients	Single Center	396 patients (318 HCC, 78 ICC)	49 patients (40 HCC, 9 ICC)	49	Contrast CT/portal venous	Pathology diagnosis after surgery
Liu et al. [16]	2023	China	Retrospective Cohort	April 2016–December 2021.	HCC patients	Single Center	124	53	53	MRI/arterial and portal venous phase	Histopathology proven
Zhang et al. [17]	2023	China	Retrospective Case–Control	-	HCC patients	Single Center	222	-	95	Contrast CT/arterial and portal venous phase	Manual label for venous phase images
Midya et al. [31]	2018	USA	Retrospective Cohort	2000–2015	HCC patients	Single Center	156	27	156	Contrast CT/portal venous	Histopathology proven
Xie et al. [7]	2025	China	Retrospective Cohort	May 2008 to January 2024	Ternary classification of hepatocellular carcinoma (HCC) and Hepatic Inflammatory Pseudotumor (HIPT)	Multicenter	196	-	84	DCE-MRI/(arterial, portal venous, delayed phases)	Histopathology proven
Wang et al. [18]	2025	China	Retrospective	-	Hepatic Inflammatory and Pseudotumors (IPT)	Multicenter	112	-	146	CT contrast scan/multiphase (SOFT, AP, PVP, DP)	Histopathological examination from surgical resection
Cheng et al. [19]	2025	China	Retrospective	January 2016–October 2023	Oher primary liver cancers (non-iCCA: HCC and cHCC-iCCA) within PLC.	Single Center	124		178	CT (non-contrast) and MRI scan/ MRI: T1, T2, DWI, CE-MRI (arterial, venous, 3-min delayed)	Histopathological examination (from surgical resection or biopsy)
Wei et al. [20]	2024	China	Retrospective cohort	June 2012–December 2012	Multiclass liver pathologies (6 types)	Multicenter	1580	-	130M8	CT contrast scan/arterial and portal venous phase	Histopathology/radiologist consensus
Wang et al. [21]	2024	China	Retrospective Cohort	-	HCC patients	Single Center	113	49	162	MRI/delayed phase	Histopathology
Chen et al. [22]	2024	China	Retrospective Cohort	January 2017–September 2020	HCC and cHCC-ICC patients	Single Center	332	83	50	Single B-mode Ultrasound (BMUS) image	Histopathology (post-resection)
Midya et al. [30]	2023	USA	Retrospective Cohort	2003–2015	HCC, CRLM, and benign liver tumor patients	Multicenter	488 scans	162 scans	814 scans	CT contrast scan/portal venous phase	Histopathology (resection)
Mahmoudi et al. [32]	2023	Germany	Retrospective Cohort	2014–2021	HCC patients	Single Center	65	-	94	CT contrast scan/arterial phase	Histopathology
Hu et al. [23]	2022	China	Retrospective Cohort	2008- 2018	HCC patients	Multicenter	344	97	-	MRI scan T1C and T2W/arterial and portal venous phase	Histopathology
Huang et al. [24]	2022	China	Retrospective Case Control	October 2019–December 2021	HCC patients	Single Center	123	51	-	MRI scan T2 WI	Histopathology (surgery/biopsy)
Xu et al. [25]	2022	China	Retrospective Cohort	August 2018–November 2019	HCC patients	Single Center	122	89	211	Non-contrast CT	Histopathology
Ren et al. [26]	2021	China	Retrospective Cohort	January 2019–March 2021	HCC patients	Multicenter	149	77	187	Ultrasound (grayscale)	Histopathology
Xue et al. [27]	2021	China	Retrospective Cohort	Jan uary 2005–June 2020	Inflammatory mass with hepatolithiasis patients	Single Center	110	35	-	CT contrast scan/arterial and portal venous phase	Histopathology
Nakai et al. [33]	2021	Japan	Retrospective Cohort	January 2004–September 2019	HCC patients	Single Center	493	62	617	Non contrast CT scan/late-arterial (23 s post-trigger) and delayed (80 s post-trigger) phase	Histopathology (surgical specimen)
Xue et al. [28]	2021	China	Retrospective Cohort	January 2005–July 2019	Inflammatory mass with hepatolithiasis patients	Single Center	96	35	-	CT contrast scan/arterial phase	
Xu et al. [29]	2021	China	Retrospective Cohort	2005–2019	Inflammatory mass with hepatolithiasis patients	Multicenter	96	35	96	CT contrast scan/arterial phase	

**Table 2:** Algorithms and model characteristics.

First Author	ROI	Preprocessing	Model Structure	AI Classifier	Validation	Comparison Algorithms
Xiong et al. [15] (2025)	Manual delineation of tumor boundary by an experienced radiologist, followed by cropping of the minimum rectangular region containing the tumor	ROI images resized to 224 × 224 pixels; data augmentation (rotation, flip) applied to the minority class (ICC) to address imbalance	ConvNeXt-ECSAM (ConvNeXt-T backbone with proposed ECSAM attention modules, fully connected layer for classification)	2D CNN	Internal hold-out validation (8:1:1 split on patient level)	Compared with ResNet152, DenseNet121, Vision Transformer, Swin Transformer
Liu et al. [16] (2023)	Manual segmentation of the entire tumor volume on axial FS-T2WI, AP, and PVP images by a radiologist	Image segmentation, feature extraction using IBEX software (β1.0), Z-score normalization of features	Logistic regression model combining selected radiomics features and clinical risk factors	Radiomics (machine learning–logistic regression)	Internal hold-out validation (7:3 split on patient level)	Compared with individual sequence radiomics models (FS-T2WI, AP, PVP), joint radiomics model (JR), and clinical model (C)
Zhang et al. [17] (2023)	Automated segmentation of liver lesion using a modified 2D U-Net, followed by cropping a fixed-size 3D volume (256 × 256 × 36 voxels) around the centroid	Windowing transformation (arterial: [−50, 130] HU; venous: [−45, 205] HU), intensity normalization	3D dense convolutional network with dual-branch architecture (arterial and venous), followed by a feature fusion network	3D CNN	Internal hold-out validation (7:3 split on patient level)	Compared with arterial sub-network and venous sub-network alone
Midya et al. [31] (2018)	Semi-automated segmentation (Scout Liver) supervised by expert radiologist	Normalization [0, 1], intensity threshold −100 HU to 300 HU, cropped to largest tumor, resized to 299 × 299 pixels	Modified Inception-v3; last 4 layers removed; added 3 fully connected layers with 7000, 1024, 1 nodes; ReLU activations; final SoftMax output	Deep convolutional neural network (modified Inception-v3)	Random split: 70% training, 30% test	No
Xie et al. [7] (2025)	Sequences: T2WI, DCE-MRI (arterial, portal venous, delayed phases), DWI	Manual 3D lesion segmentation on ITK-SNAP	Resampling, min–max normalization (0–1), Z-score feature standardization	Logistic regression	Machine learning (radiomics)	Compared with radiomics-only, clinical-only, fusion model
Wang et al. [18] (2025)	Manual segmentation of the entire lesion slice-by-slice by two radiologists (>5 yrs experience) using ITK-SNAP, fine-tuned by senior radiologist (>15 yrs)	Spatial matching of ROI to original image, window width/level normalization.	14 ML models tested (CatBoost, LightGBM, LR, NB, LDA, QDA, KNN, GBC, XGBoost, RF, AdaBoost, ET, DT). Optimal fused model: Linear Discriminant Analysis (LDA)	Machine learning (PyRadiomics feature extraction), LASSO for feature selection, 5-fold CV with grid search for hyperparameter tuning	Internal: 5-fold cross-validation on training set; hold-out test: 70/30 split (training *n* = 102/test *n* = 44)	Performance compared among clinical features alone, radiomic features alone, and fused radiomic + clinical features
Cheng et al. [19] (2025)	Manual segmentation on largest tumor slice	Resampling to 1 × 1 × 1 mm^3^, bicubic spline interpolation	ResNet-50 (transfer learning)	Deep learning (ResNet-50), PCA, LASSO, logistic regression	10-fold cross-validation (training); hold-out test (70/30 split)	Intra-modality: DLRS vs. DLRR vs. radiological model; inter-modality: CT vs. MRI; fused CT-MRI model
Wei et al. [20] (2024)	Automatic (YOLOv8 + 3D liver segmentation)	Cropping, normalization, augmentation	ResNet50 + self-supervised pretext + SKD	Deep CNN (ResNet50-based)	Internal test, external validations	Yes (vs. radiologists)
Wang et al. [21] (2024)	Original tumor ROI ± expanded regions (−2 to +8 mm)	Resampling to 224 × 224, adaptive histogram equalization, normalization	ResNet50 (pre-trained) for feature extraction + SVM classifier	Deep learning (ResNet50) + machine learning (SVM)	Internal 70:30 split	No
Chen et al. [22] (2024)	Single optimal slice showing maximum tumor diameter and details	Data augmentation (rotation, cropping, translation)	ResNet18 (17 convolutional layers, 1 fully connected layer, 4 residual blocks); end-to-end classification	Deep learning (ResNet18)	5-fold cross-validation on training set, final evaluation on independent test cohort	Vs. MobileNet, DenseNet121, Inception V3
Midya et al. [30] (2023)	Semi-automated tumor segmentation (Scout Liver software (Analogic Corporation, Peabody, MA, USA))	Thresholding (−100 to 300 HU), normalization (0–1), resizing to 299 × 299 px	Modified Inception v3 (final layers replaced with FC layers: 7000, 1024, 4 nodes)	Deep learning (CNN–transfer learning)	Hold-out (60/20/20 split)	Yes (vs. radiologists, VGG, ResNet, DenseNet, Inception v3)
Mahmoudi et al. [32] (2023)	Three spherical VOIs (1 cm diameter) in hypervascular tumor region	StandardScaler normalization, LASSO feature selection	Logistic regression classifier	Machine learning (radiomics)	Hold-out (70% train, 30% test)	Yes (vs. AdaBoost, Stochastic GB, random forest)
Hu et al. [23] (2022)	Manual segmentation (3D Slicer)	Feature extraction (IBSI), min–max scaling	TPOT AutoML pipeline	Genetic programming (AutoML)	Hold-out test set	Radiologist performance
Huang et al. [24] (2022)	Manual segmentation on largest lesion slice (ITK-SNAP)	ICC > 0.8 for feature stability, mRMR + LASSO for selection	Radiomics nomogram (LASSO + logistic regression)	Radiomics signature	Internal hold-out (7:3 split)	-
Xu et al. [25] (2022)	Manual segmentation (3D Slicer) by radiologists	Voxel spacing standardized to 1 × 1 × 1 mm, intensity discretization (bin width = 25 HU)	Feature extraction (Pyradiomics) → feature selection (LASSO) → SVM classification	Support vector machine (SVM)	Internal validation (split-sample)	Radiomics model vs. radiologist evaluation
Ren et al. [26] (2021)	Manual segmentation (ITK-SNAP) by experienced radiologists	Normalization, resampling to 1 × 1 mm, gray-level discretization (bin width = 25)	Feature extraction (Pyradiomics) → feature selection (variance filter + LASSO) → SVM classification	Support vector machine (SVM)	Internal test (*n* = 38) + external validation (*n* = 39)	Combined vs. clinical vs. ultrasonics-only models
Xue et al. [27] (2021)	Manual segmentation of tumor (2 radiologists)	Windowing (W:200, L:45 HU), resampling to 512 × 512, uniform slice thickness 5 mm	Radiomic signature via LASSO logistic regression combining rad score from portal venous and arterial phase	Radiomics (LIFEx)	External validation (second affiliated hospital, *n* = 35)	No
Nakai et al. [33] (2021)	Manual cropping (RectLabel) on representative axial slice ± neighboring slices (9 images/case)	Intensity truncation (–125 to 225 HU), resizing to 70 × 70, normalization (mean = 0, SD = 1)	Custom 3D CNN: 4 convolutional blocks (Conv + ReLU + MaxPool) → fully connected layers (100, 100, 30, 3 nodes)	Convolutional neural network (CNN)–PyTorch (version 1.5.0)	Hold-out test set (*n* = 62)	One-input model (CT only) vs. two-input model (CT + markers) vs. radiologists
Xue et al. [28] (2021)	Manual segmentation of tumor (2 radiologists)	Windowing (W:200, L:45 HU), resampling to 512 × 512, uniform slice thickness 5 mm	Radiomic signature via LASSO logistic regression	Radiomics (LIFEx)	External validation (second affiliated hospital, *n* = 35)	Yes (vs. clinical model)
Xu et al. [29] (2021)	Manual segmentation of tumor mass	Window: 200 HU width, 45 HU level; pixel: 512 × 512	1. Radiomic feature extraction (52 features); 2. feature selection (LASSO); 3. model building (logistic regression)	Logistic regression (for Rad-score and comprehensive model)	External validation cohort from another hospital	Yes (radiomic vs. clinical vs. comprehensive model)

## Data Availability

The data supporting the findings of this study are available within the article and its Supplementary Materials. All data were derived from previously published studies, which are cited in the reference list.

## Supplementary Materials

The following supporting information can be downloaded at: https://www.mdpi.com/article/10.3390/cancers18060937/s1, Supplementary Table S1: Summary of sample and selected dataset characteristics. Supplementary Table S2: Summary of Radiomics Quality Score (RQS). Supplementary Figure S1: Forest Plot with pooled NLR and PLR. Supplementary Figure S2: Sensitivity Analysis using leave one-out method. Supplementary Figure S3: Forest Plot with pooled sensitivity and specificity for type of test set subgroup. Supplementary Figure S4: Forest Plot with pooled NLR and PLR for type of test set subgroup. Supplementary Figure S5: Forest Plot with pooled sensitivity and specificity for type of Validation. Supplementary Figure S6: Forest Plot with NLR and PLR for type of Validation. Supplementary Figure S7: Forest Plot with pooled sensitivity and specificity for type of AI classifier. Supplementary Figure S8: Forest Plot with NLR and PLR for type of AI classifier. Supplementary Figure S9: Forest Plot with pooled sensitivity and specificity for type of Diagnostic Modality. Supplementary Figure S10: Forest Plot with NLR and PLR for type of Diagnostic Modality. Supplementary Figure S11: Forest Plot with pooled sensitivity and specificity for type of Segmentation. Supplementary Figure S12: Forest Plot with NLR and PLR for type of Segmentation.
