# Supplementary material for: Radiomics for Detection and Differentiation of Intrahepatic Cholangiocarcinoma: A Systematic Review and Meta-Analysis
Source: Cancers (Basel). 2026 Mar 13;18(6):937. doi: 10.3390/cancers18060937 (PMC13024450; doi:10.3390/cancers18060937)

Supplementary Data

1. Search Strategy:

Database	Search query	
PubMed	("Radiomics"[Mesh] OR radiomic*[tiab] OR "texture analy*"[tiab] OR "image analy*"[tiab] OR "quantitative imaging"[tiab] OR "computational imaging"[tiab] OR "delta-radiomics"[tiab] OR "radiogenomic*"[tiab] OR "feature extraction"[tiab] OR "image biomarker*"[tiab] OR "artificial intelligence"[Mesh] OR "artificial intelligence"[tiab] OR "machine learning"[Mesh] OR "machine learning"[tiab] OR "deep learning"[tiab] OR "convolutional neural network*"[tiab] OR CNN[tiab] OR "neural network*"[tiab] OR "AI model"[tiab] OR "predictive model"[tiab]) AND ("Cholangiocarcinoma"[Mesh] OR "intrahepatic cholangiocarcinoma"[tiab] OR "intrahepatic bile duct carcinoma"[tiab] OR "intrahepatic bile duct cancer"[tiab] OR "bile duct neoplasm*"[tiab] OR ICC[tiab]) AND (diagnos*[tiab] OR "differential diagnosis"[tiab] OR classif*[tiab] OR differentiat*[tiab] OR discriminat*[tiab] OR distinguish*[tiab] OR identificat*[tiab] OR accurac*[tiab] OR performance[tiab] OR AUC[tiab]) AND ("computed tomography"[tiab] OR CT[tiab] OR "magnetic resonance imaging"[tiab] OR MRI[tiab] OR ultrasound[tiab] OR "positron emission tomography"[tiab] OR PET[tiab] OR imaging[tiab])	1139
Embase	('radiomics' OR 'texture analysis' OR 'image analysis' OR 'quantitative imaging' OR 'computational imaging' OR 'delta radiomics' OR 'radiogenomics' OR 'feature extraction':ti,ab,kw OR 'artificial intelligence' OR 'machine learning' OR 'deep learning' OR cnn OR 'ai model' OR 'predictive model') AND ('cholangiocarcinoma' OR 'intrahepatic bile duct carcinoma' OR icc) AND (diagnos* OR classif* OR differentiat* OR discriminat* OR distinguish* OR identificat* OR accurac* OR performance OR auc) AND ('computed tomography' OR ct OR 'magnetic resonance imaging' OR mri OR ultrasound OR 'positron emission tomography' OR pet OR imaging)	3,751
Scopus	TITLE-ABS-KEY(radiomic* OR "texture analy*" OR "image analy*" OR "quantitative imaging" OR "computational imaging" OR "delta-radiomics" OR "radiogenomic*" OR "feature extraction" OR "image biomarker*" OR "artificial intelligence" OR "machine learning" OR "deep learning" OR "convolutional neural network*" OR CNN OR "neural network*" OR "AI model" OR "predictive model") AND TITLE-ABS-KEY("intrahepatic cholangiocarcinoma" OR "intrahepatic bile duct carcinoma" OR "intrahepatic bile duct cancer" OR ICC) ANDTITLE-ABS-KEY(diagnos* OR classif* OR differentiat* OR discriminat* OR distinguish* OR identificat* OR accurac* OR performance OR AUC) AND TITLE-ABS-KEY(CT OR MRI OR ultrasound OR PET OR imaging)	2,945
Cochrane	("radiomics" OR "quantitative imaging" OR "computational imaging" OR "texture analysis" OR "image biomarker*" OR "feature extraction" OR "delta-radiomics" OR "radiogenomic*" OR "artificial intelligence" OR "machine learning" OR "deep learning" OR "convolutional neural network*" OR CNN OR "AI model" OR "predictive model") AND ("intrahepatic cholangiocarcinoma" OR "cholangiocarcinoma" OR ICC) AND (diagnos* OR classif* OR differentiat* OR discriminat* OR distinguish* OR identificat* OR accurac* OR performance)	97

2. Supplementary Tables

Supplementary Table S1: Summary of sample and selected dataset characteristics				
Study	Local Data Source	No of Patient (ICC/ Other liver pathology or health patients)	Male/Female	Age
Xiong et al. (2025)	First Affiliated Hospital of Nanchang University	494 (97/397)	415/79	53.9 ± 12.4
Liu et al. (2023)	Affiliated Hospital of North Sichuan Medical College (Nanchong, China).	177 (48/129)	131/46	-
Zhang et al. (2023)	Shanghai Eastern Hepatobiliary Hospital	317 (171/146)	-	-
Midya et al. (2018)	-	223 (107/116))	-	-
Xie et al. (2025)	Three hospitals: Ganzhou People's Hospital, Nanfang Hospital, Southern Medical University	280 (80/200)	218/62	HCC: 54.80 ± 11.83 ICC: 59.78 ± 9.21 HIPT: 56.70 ± 13.47
Wang et al. (2025)	-	146 (112/34)	86/60	54.12 ± 11.19
Cheng et al. (2025)	The First Affiliated Hospital of Zhengzhou University	178 (39/139)	124 / 54	-
Wei et al. (2024)	Henan Provincial People's Hospital (HN)<br>First Affiliated Hospital of Chengdu Medical College (CD)<br>Leshan People's Hospital (LS)<br>Guizhou Provincial People's Hospital (GZ)<br>West China Tianfu Center<br>Sanya People's Hospital	4039	-	Internal Training set: ICC (n = 323)-Age: 57.16 ± 12.10 years, Internal Test set: ICC (n = 80)-Age: 57.27 ± 12.28 year

Wang et al. (2024)	Fifth Medical Center of Chinese PLA General Hospital	162 (75/87)	112/ 50	-
Chen et al. (2024)	-	465 (105/360)	351/114	HCC: 57.4 ± 11.2. ICC: 61.5 ± 11.5. cHCC-ICC: 57.1 ± 11.1.
Midya et al. (2023)	Memorial Sloan Kettering Cancer Center, Washington University, Erasmus Medical Center	814 scans (246/568)	-	-
Mahmoudi et al. (2023)	University Hospital Frankfurt	94 (47/47)	68/26	63.3 ± 12.4
Hu et al. (2022)	Second Xiangya Hospital, Hospital of the University of Pennsylvania, and Rhode Island Hospital	489 (207/282)	-	-
Huang et al. (2022)	Hunan Provincial People’s Hospital	174 (61/113)	138/36	HCC: 54.9 ± 11.4 ICC: 58.4 ± 10.4
Xu et al. (2022)	Affiliated Drum Tower Hospital, Nanjing	211 (53/158)	154/57	-
Ren et al. (2021)	3 Hospitals in Henan (Provincial People’s Hospital, Cancer Hospital, First Affiliated Hospital of Zhengzhou)	226 (50/176)	163/63	58.3 ± 10.5 years
Xue et al. (2021)	Wenzhou Medical University	145 (61/84)	90/55	-
Nakai et al. (2021)	Kyoto University Hospital	617 (122/495)	-	67.9 ± 10.1
Xue et al. (2021)	First Affiliated Hospital of Wenzhou Medical University	131 (53/78)	80/51	-
Xu et al. (2021)	The First Affiliated Hospital of Wenzhou Medical University	131 (53/78)	Training: 59/37 Validation: 17/13	-

Supplementary Table S2: Summary of Radiomics Quality Score (RQS)

**RQS Item**	**Image protocol quality**	**Multiple segmentations**	**Phantom study**	**Imaging at multiple time points**	**Feature reduction or adjustment for multiple testing**	**Multivariable analysis**	**Detect and discuss biological correlates**	**Cut-off analyses**	**Discrimination statistics**	**Calibration statistics**	**Prospective study**	**Validation**	**Comparison to 'gold standard'**	**Potential clinical utility**	**Cost-effectiveness analysis**	Open science and data	TOTAL RQS SCORE
Xiong 2025	Protocols Well Documented	No	No	No	Either measure is implemented	No	No	No	A discrimination statistic and its statistical significance are reported;A resampling method technique is also applied	None	No	validation is based on a dataset from the same institute	Yes	No	No	scans are open source	11/36
Liu 2023	Protocols Well Documented	Yes	No	No	Either measure is implemented	Yes	Yes	No	A discrimination statistic and its statistical significance are reported;A resampling method technique is also applied	a resampling method technique is applied	No	validation is based on a dataset from the same institute	Yes	No	No	scans are open source+ radiomics features are calculated on a set of representative ROIs and the calculated features and representative ROIs are open source	16/36
Zhang 2023	Protocols Well Documented	Yes	No	No	Either measure is implemented	No	No	No	A discrimination statistic and its statistical significance are reported;A resampling method technique is also applied	None	No	validation is based on a dataset from the same institute	Yes	No	No	Not Provided	11/36
Midya 2018	Protocols Well Documented	No	No	No	Either measure is implemented	No	No	No	A discrimination statistic and its statistical significance are reported;A resampling method	None	No	validation is based on a dataset from the same institute	Yes	No	No	scans are open source+ radiomics features are calculated on a set of representative ROIs and the calculated	12/36

									technique is also applied							features and representative ROIs are open source	
Xie 2025	Protocols Well Documented	Yes	No	No	Either measure is implemented	No	No	No	A discrimination statistic and its statistical significance are reported;A resampling method technique is also applied	None	No	validation is based on a dataset from another institute	Yes	No	No	scans are open source	13/36
Wang 2025	None	Yes	No	No	Either measure is implemented	Yes	No	No	A discrimination statistic and its statistical significance are reported;A resampling method technique is also applied	None	No	validation is based on a dataset from the same institute	Yes	No	No	scans are open source+ radiomics features are calculated on a set of representative ROIs and the calculated features and representative ROIs are open source	13/36
Cheng 2025	Protocols Well Documented	Yes	No	No	Either measure is implemented	Yes	Yes	No	A discrimination statistic and its statistical significance are reported;A resampling method technique is also applied	None	No	validation is based on a dataset from the same institute	Yes	No	No	scans are open source+ radiomics features are calculated on a set of representative ROIs and the calculated features and representative ROIs are open source	15/36
Wei 2024	Protocols Well Documented	Yes	No	No	Neither measure is implemented	No	No	No	A discrimination statistic and its statistical significance are reported;A resampling method technique is also applied	None	Yes	validation is based on three or more datasets from distinct institutes	Yes	Yes	No	scans are open source+ radiomics features are calculated on a set of representative ROIs and the calculated features and representative ROIs are open source+ the code is open sourced+ region of interest segmentations are open source	24/36
Wang 2024	Protocols Well Documented	Yes	No	No	Either measure is implemented	No	No	No	A discrimination statistic and its statistical significance are reported;A resampling method technique is also applied	None	No	validation is based on a dataset from the same institute	Yes	No	No	Not Provided	11/36
Chen 2023	Protocols Well Documented	Yes	No	No	Either measure is implemented	Yes	Yes	No	A discrimination statistic and its statistical significance are reported;A resampling method technique is also applied	None	No	validation is based on a dataset from the same institute	Yes	No	No	scans are open source	14/36
Midya 2023	Protocols Well Documented	Yes	No	No	Either measure is implemented	No	No	No	A discrimination statistic and its statistical	None	No	validation is based on a dataset from	Yes	No	No	Not Provided	12/36

									significance are reported;A resampling method technique is also applied			another institute					
Mahmoudi 2022	Protocols Well Documented	Yes	No	No	Either measure is implemented	No	No	Yes	A discrimination statistic and its statistical significance are reported;A resampling method technique is also applied	None	No	validation is based on a dataset from the same institute	Yes	No	No	scans are open source	13/36
Hu 2022	Protocols Well Documented	Yes	No	No	Either measure is implemented	No	No	Yes	A discrimination statistic and its statistical significance are reported;A resampling method technique is also applied	None	No	validation is based on a dataset from the same institute	Yes	No	No	scans are open source+ radiomics features are calculated on a set of representative ROIs and the calculated features and representative ROIs are open source	14/36
Huang 2022	Protocols Well Documented	Yes	No	No	Either measure is implemented	Yes	Yes	No	A discrimination statistic and its statistical significance are reported;A resampling method technique is also applied	None	No	validation is based on a dataset from the same institute	Yes	No	No	Not Provided	13/36
Xu 2021	Protocols Well Documented	Yes	No	No	Either measure is implemented	Yes	Yes	No	A discrimination statistic and its statistical significance are reported;A resampling method technique is also applied	None	No	validation is based on a dataset from the same institute	Yes	No	No	scans are open source	14/36
Ren 2021	Protocols Well Documented	Yes	No	No	Either measure is implemented	Yes	Yes	No	A discrimination statistic and its statistical significance are reported;A resampling method technique is also applied	None	No	validation is based on a dataset from the same institute	Yes	No	No	scans are open source+ radiomics features are calculated on a set of representative ROIs and the calculated features and representative ROIs are open source	15/36
Xue 2021	Protocols Well Documented	No	No	No	Either measure is implemented	Yes	Yes	No	A discrimination statistic and its statistical significance are reported;A resampling method technique is also applied	None	No	validation is based on a dataset from the same institute	Yes	No	No	scans are open source+ radiomics features are calculated on a set of representative ROIs and the calculated features and representative ROIs are open source+ the code is open sourced	17/36
Nakai 2021	Protocols Well Documented	Yes	No	No	Either measure is implemented	No	Yes	No	A discrimination statistic and its statistical significance are reported;A	None	No	No validation	Yes	No	No	scans are open source+ radiomics features are calculated on a set of representati	12/36

									resampling method technique is also applied							ve ROIs and the calculated features and representative ROIs are open source	
Xue 2021	Protocols Well Documented	Yes	No	No	Either measure is implemented	Yes	Yes	No	A discrimination statistic and its statistical significance are reported;A resampling method technique is also applied	None	No	validation is based on a dataset from the same institute	Yes	Yes	No	scans are open source+ radiomics features are calculated on a set of representative ROIs and the calculated features and representative ROIs are open source	17/36
Xu 2021	Protocols Well Documented	No	No	No	Either measure is implemented	No	No	No	A discrimination statistic and its statistical significance are reported;A resampling method technique is also applied	None	No	validation is based on a dataset from the same institute	Yes	No	No	scans are open source	11/36

3. Supplementary Figures

Supplementary Figure S1: Forest Plot with pooled NLR and PLR

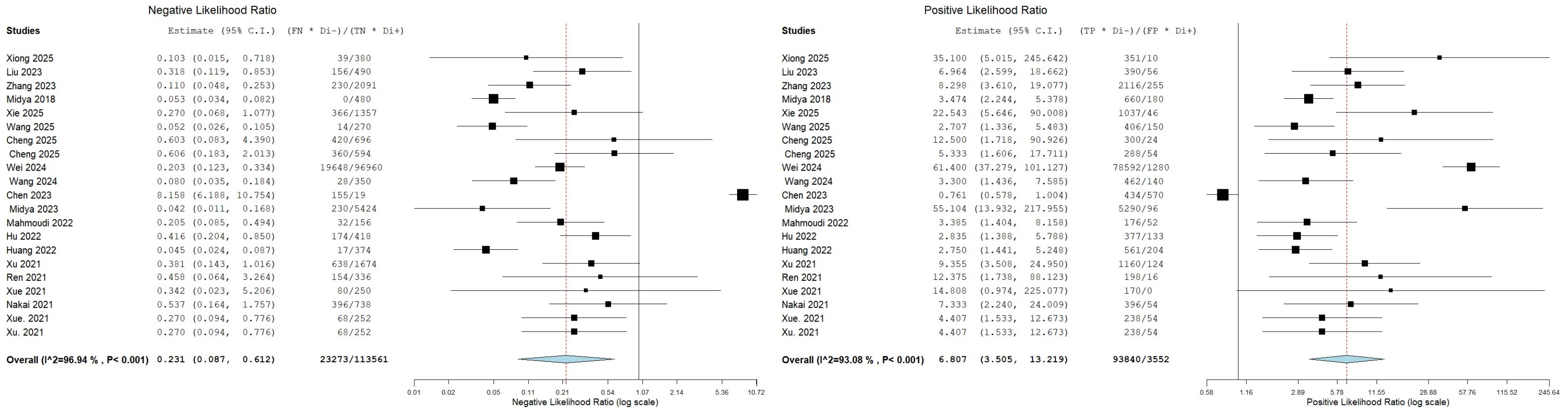

Supplementary Figure S2: Sensitivity Analysis using leave one-out method

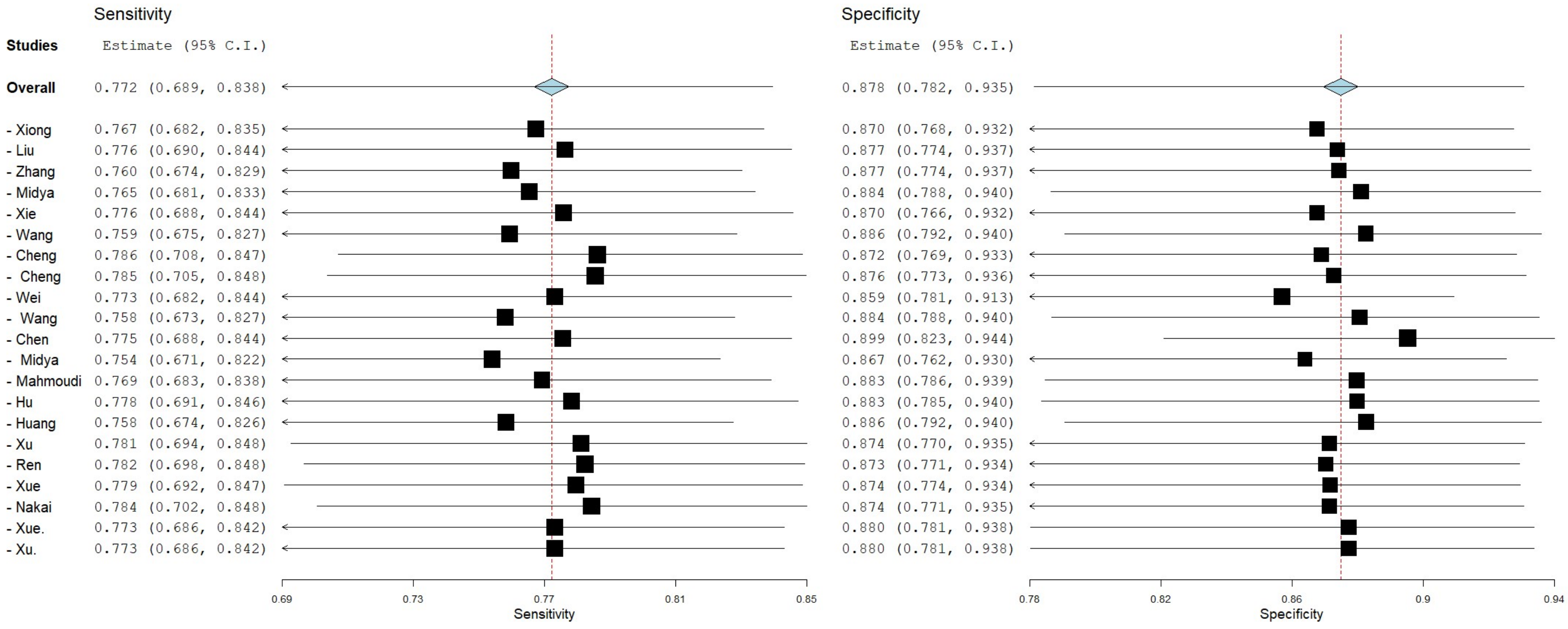

Supplementary Figure S3: Forest Plot with pooled sensitivity and specificity for type of test set subgroup

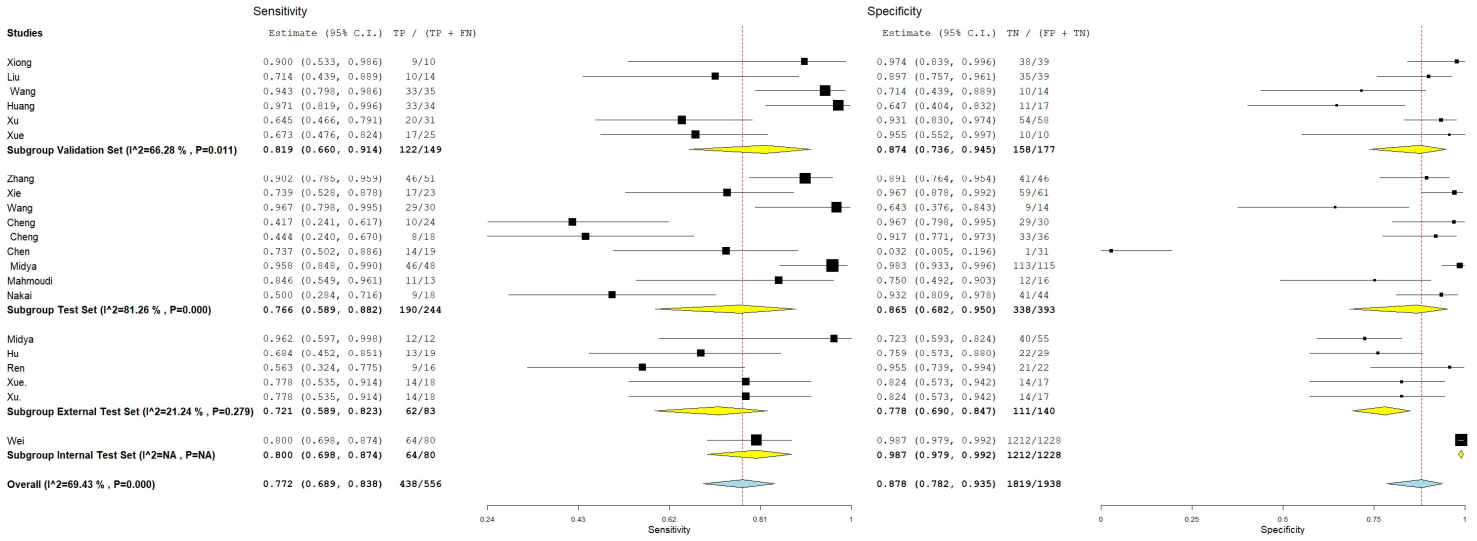

Supplementary Figure S4: Forest Plot with pooled NLR and PLR for type of test set subgroup

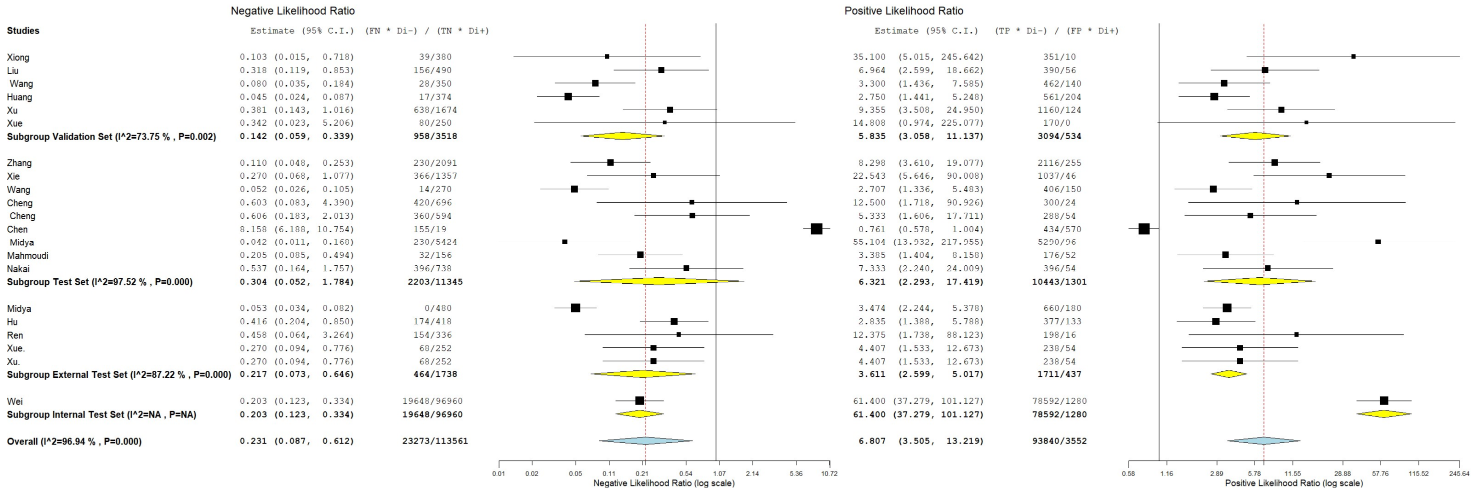

Supplementary Figure S5: Forest Plot with pooled sensitivity and specificity for type of Validation

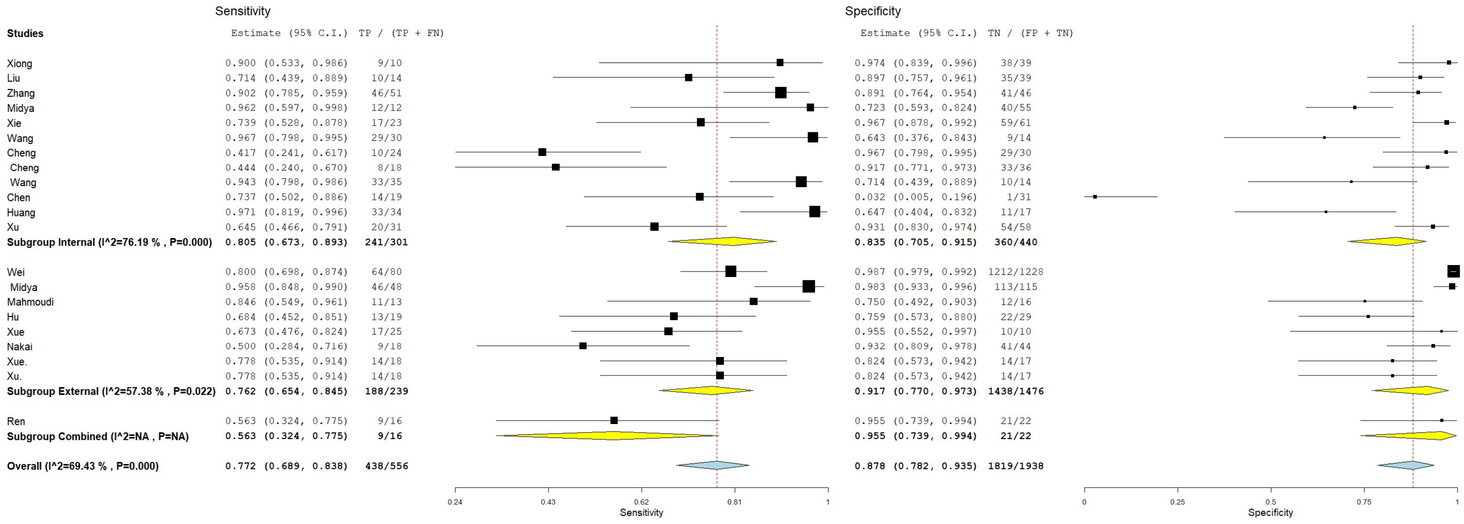

Supplementary Figure S6: Forest Plot with NLR and PLR for type of Validation

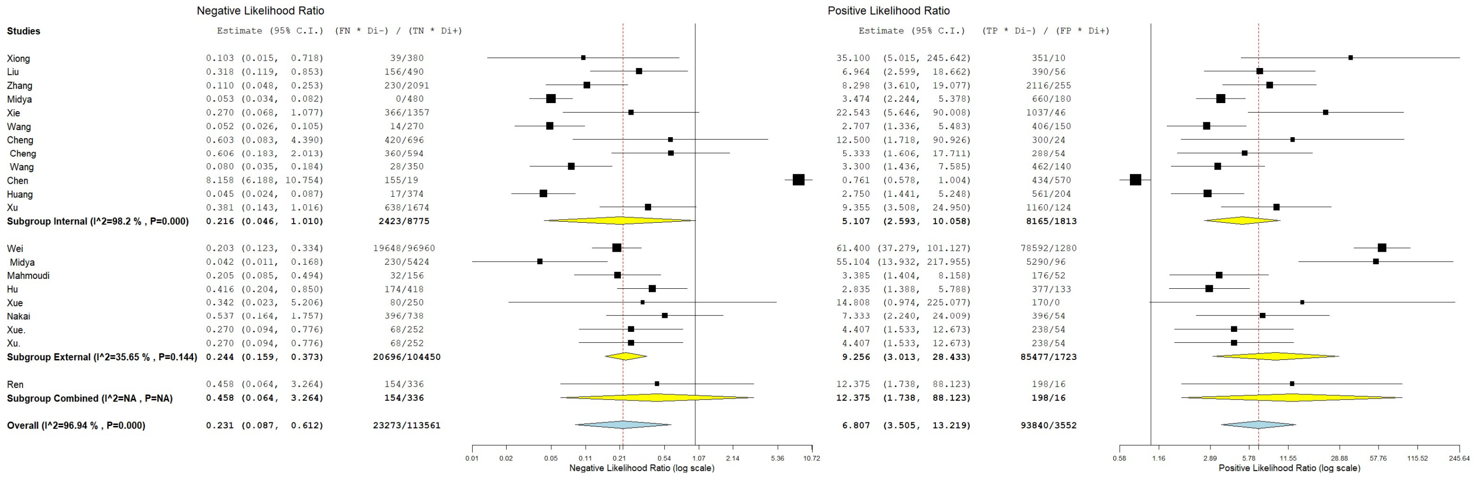

Supplementary Figure S7: Forest Plot with pooled sensitivity and specificity for type of AI classifier

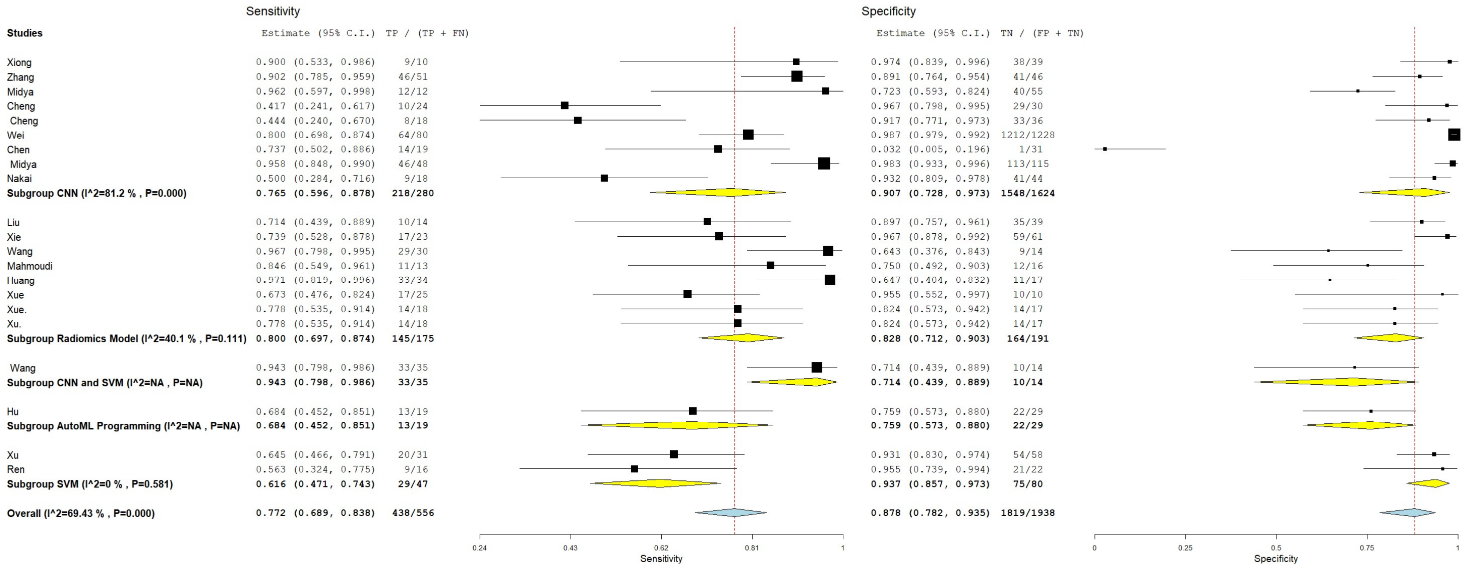

Supplementary Figure S8: Forest Plot with NLR and PLR for type of AI classifier

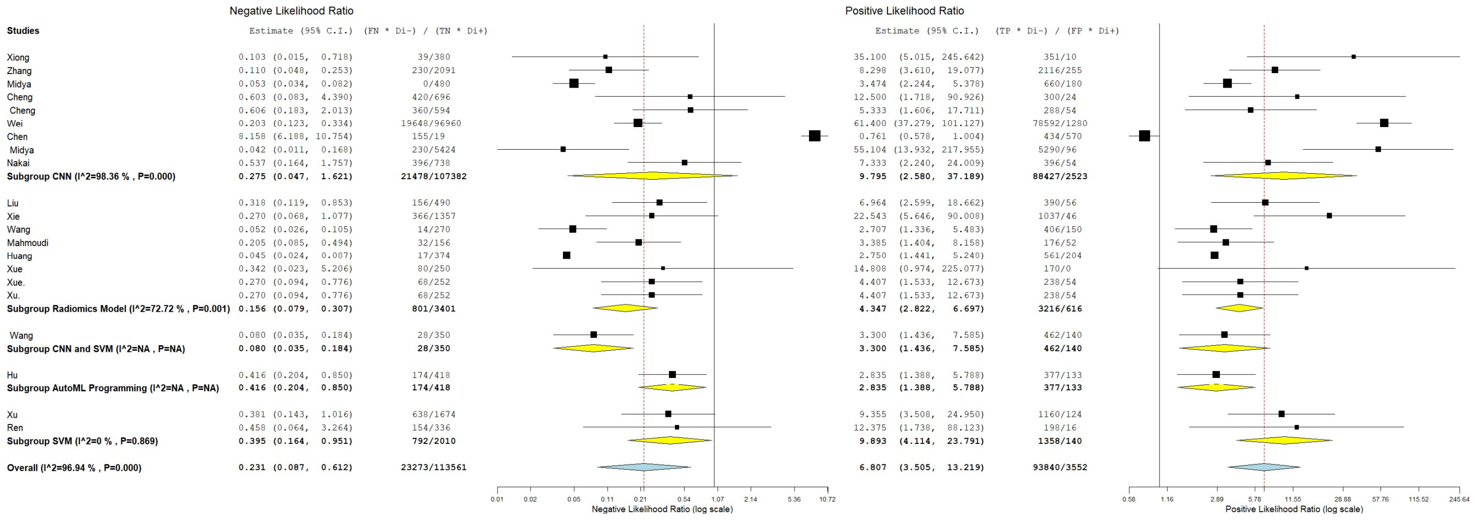

Supplementary Figure S9: Forest Plot with pooled sensitivity and specificity for type of Diagnostic Modality

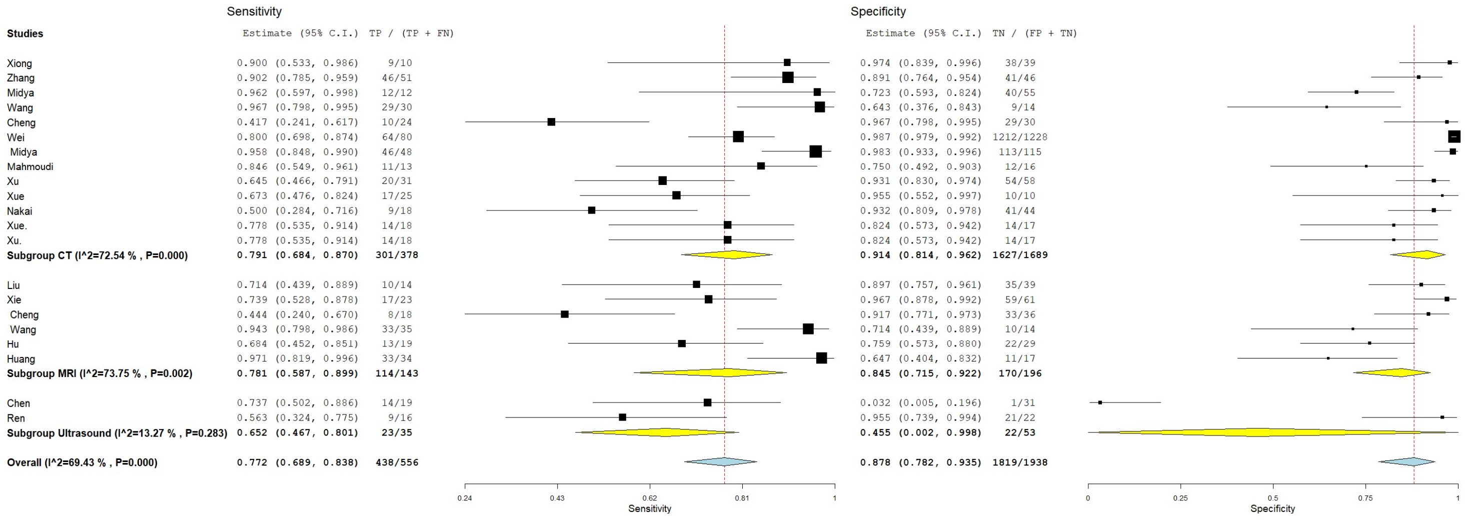

Supplementary Figure S10: Forest Plot with NLR and PLR for type of Diagnostic Modality

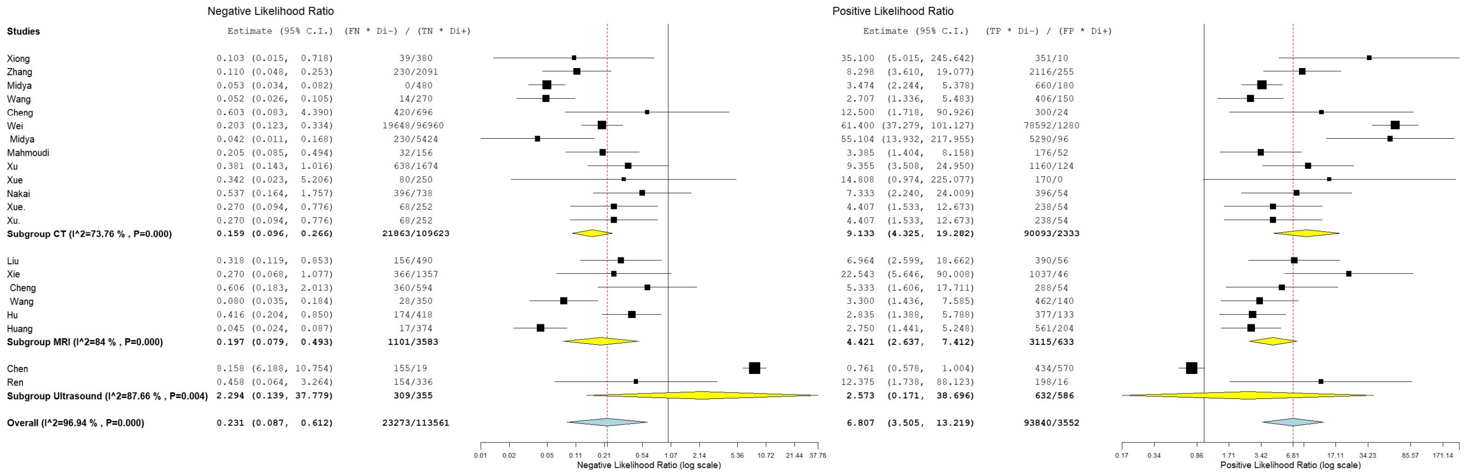

Supplementary Figure S11: Forest Plot with pooled sensitivity and specificity for type of Segmentation

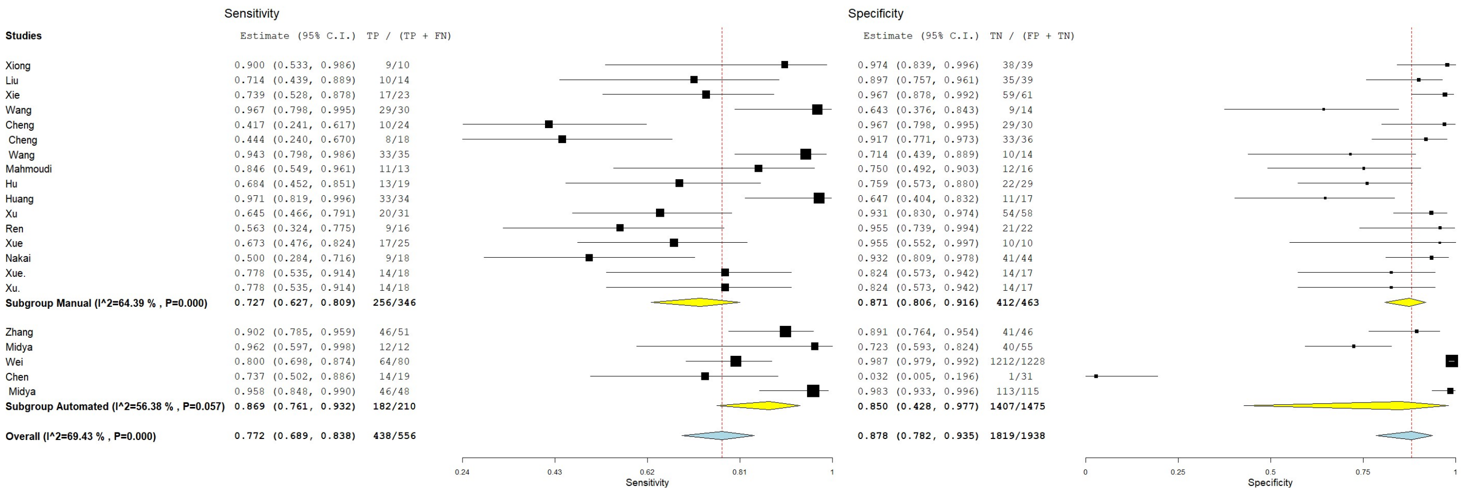

Supplementary Figure S12: Forest Plot with NLR and PLR for type of Segmentation

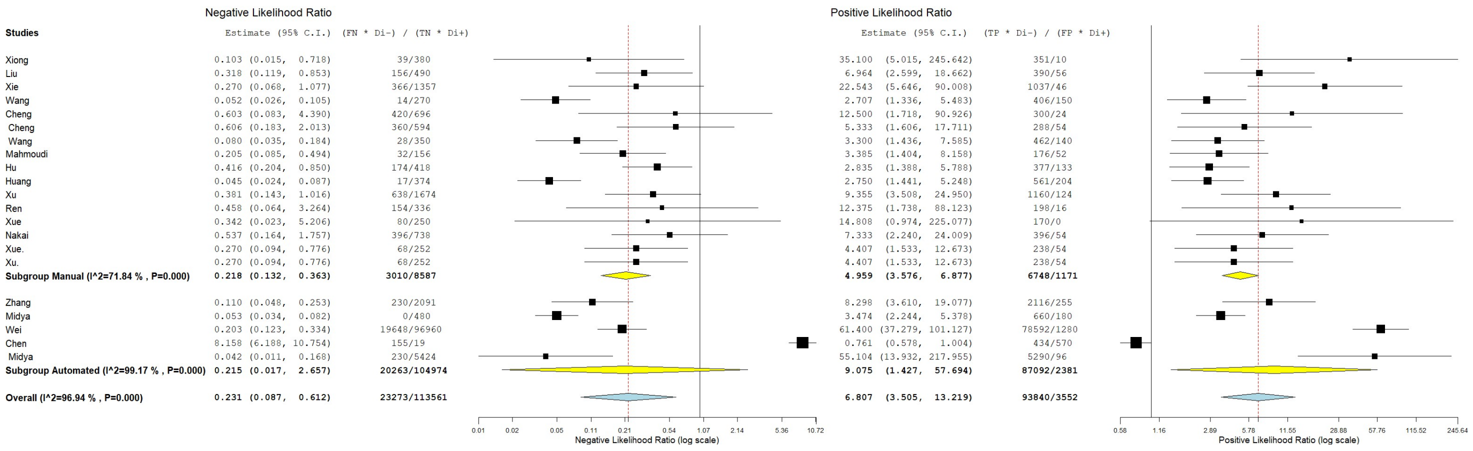

Supplement: Supplementary file 1 [file cancers-18-00937-s001.zip › cancers-4184828-supplementary.pdf]
